# Species Diversity of Edible Mushrooms VI: Morphological and Phylogenetic Evidence Supports Five New Species of *Laccaria* (Hydnangiaceae, Agaricales) from China and Thailand

**DOI:** 10.3390/jof12080618

**Published:** 2026-08-17

**Authors:** Guo Zhao, Shu-Juan Tang, Ya-Ting Kong, Ting-Ting Liu, Hong-Wei Shen, Song-Ming Tang, Zong-Long Luo

**Affiliations:** 1 College of Agriculture and Biological Science, Dali University, Dali 671003, China; zhaoguo02@outlook.com (G.Z.); 18796932833@163.com (S.-J.T.); 18313500357@163.com (Y.-T.K.); ting060623@outlook.com (T.-T.L.); hongweifungi@outlook.com (H.-W.S.); 2 Co-Innovation Center for Cangshan Mountain and Erhai Lake Integrated Protection and Green Development of Yunnan Province, Dali University, Dali 671003, China

**Keywords:** five novel species, macrofungi, multigene, taxonomy, phylogeny

## Abstract

*Laccaria* is a cosmopolitan genus of ectomycorrhizal fungi that plays an important role in forest ecosystems worldwide. To explore the species diversity of *Laccaria* in Asia, this study conducted morphological observations and multi-gene phylogenetic analyses on 12 specimens collected from China and Thailand. The results showed that these 12 specimens formed five distinct and well-supported clades, which, combined with morphological comparisons, were recognized as five new species: *L. aurantirufescens*, *L. bisporus*, *L. jilongensis*, *L. longicystidiata*, and *L. yadongensis*. The main diagnostic characteristics of each new species are as follows: *L. aurantirufescens* has small to medium-sized basidiomata, an orange-red to russet pileus, and a yellowish-brown to reddish-brown stipe surface; *L. bisporus* has small basidiomata, a pale orange to orange-white pileus, and a brown to yellowish-brown stipe surface, and it possesses cheilocystidia, pleurocystidia, and caulocystidia; *L. jilongensis* has large basidiomata, with a pileus reaching up to 11 cm in diameter, orange-red coloration, sparse lamellae, a stipe up to 15 cm long, and the presence of cystidia; *L. longicystidiata* has small basidiomata, a pale orange to grayish-orange pileus, a light brown to brown stipe surface, and long cheilocystidia; *L. yadongensis* has small to medium-sized basidiomata, a pinkish-brown to fleshy-brown pileus that is darker at the center, four-spored basidia with sterigmata extending up to 10 μm in length, and a pileipellis composed of inflated hyphae, some of which exhibit brown pigmentation. These characteristics distinguish them from their closely related species. These findings not only enhance our understanding of *Laccaria* species diversity in Asia but also highlight the increasingly important role that Asia is poised to play in global fungal diversity conservation and taxonomic research.

## 1. Introduction

As a widely distributed group of ectomycorrhizal fungi [1], species of the genus *Laccaria* form symbiotic associations with a diverse range of host plants, thereby playing indispensable ecological roles in forest ecosystems worldwide [2,3,4,5,6,7,8]. These symbiotic interactions facilitate nutrient exchange between fungi and their host trees, promote plant nutrient uptake, and contribute to the stability of forest communities. Given their considerable ecological significance, *Laccaria* species have attracted extensive taxonomic and evolutionary research interest globally.

The genus *Laccaria* (Hydnangiaceae Gäum. & C.W. Dodge, Agaricales Underw, Agaricales) was formally established by Berkeley and Broome [9]. It is a cosmopolitan ectomycorrhizal genus, with more than 130 species described worldwide to date [10,11,12,13,14,15,16,17,18,19,20,21,22]. Morphologically, *Laccaria* species are generally recognizable by the following features: basidiomata brown, orange-yellow, or purple, with a collybioid to omphaloid habit; pileus convex, plane, or umbilicate and hygrophanous; lamellae thick and often widely spaced; basidiospores echinulate, subglobose to globose (rarely cylindrical in some species); basidia clavate, mostly four-spored, rarely one- or two-spored; cystidia sinuous or occasionally branched; and clamp connections present [23,24,25,26,27,28].

Early taxonomic studies of *Laccaria* relied predominantly on morphological observations. Owing to the paucity of molecular data and the limitations of microscopic resolution, morphological identification often resulted in ambiguous interpretations of diagnostic characteristics, leading to persistent controversies in species delimitation [23,25,29,30,31,32]. Moreover, many species within this genus exhibit highly convergent morphological traits, making it difficult to reliably delineate species boundaries based solely on phenotypic characteristics—a limitation that underscores the necessity of incorporating molecular evidence for species discrimination.

The application of molecular phylogenetic approaches has greatly advanced our understanding of this genus. Early molecular studies confirmed that *Laccaria*, *Hydnangium* Wallr., and *Podohydnangium* G.W. Beaton, Pegler & T.W.K. Young constitute a monophyletic clade [33,34,35,36,37,38]; however, the fine-scale phylogenetic relationships within this clade remained unresolved. Subsequently, Matheny et al. transferred *Laccaria* from Tricholomataceae R. Heim ex Pouzar to Hydnangiaceae based on multi-gene phylogenetic analyses [39]. Later studies further revealed that many morphospecies actually correspond to multiple cryptic phylogenetic species [40]. Wilson et al. subsequently employed ITS, LSU, *rpb*2, and *tef*1-α sequences to elucidate the origin and evolutionary history of *Laccaria* [41]. With the widespread adoption of DNA barcoding, the cryptic species diversity hidden within morphologically similar lineages of *Laccaria* has been progressively uncovered.

In Asia, systematic taxonomic research on *Laccaria* has developed rapidly despite a relatively late start. Early species identification in this region relied heavily on morphological characteristics, leading to the frequent misidentification of many Asian collections as European or North American widespread species [42]. In 2004, Wang et al. conducted the first systematic survey of *Laccaria* in southwestern China and reported two new species. Subsequently, with the application of molecular phylogenetic methods, researchers have progressively revealed the considerable cryptic diversity of the genus, successively reporting multiple new taxa including *L. acanthospora* A.W. Wilson & G.M. Muell, *L. aurantia* Popa, Rexer, Donges, Zhu L. Yang & G. Kost, and *L. yunnanensis* Popa, Rexer, Donges, Zhu L. Yang & G. Kost. Later investigations focusing on southern China and South Korea further expanded the species inventory of *Laccaria* in East Asia [18,25,27,28,42,43,44]. To date, 35 species of *Laccaria* have been recorded from southwestern China alone, whereas species inventories from Thailand and adjacent tropical Asian regions remain fragmentary [13,23,25,45,46,47]. Given that China and Thailand are situated in subtropical–temperate and tropical climatic zones, respectively, their distinct habitats may harbor substantially higher species diversity than currently recognized. However, the uneven sampling across these regions has severely hampered a comprehensive assessment of *Laccaria* diversity in Asia, highlighting the urgent need for in-depth integrative taxonomic studies.

To further explore the diversity and taxonomic relationships of *Laccaria* in Asia, we collected 12 specimens from different regions of China and Thailand. These collections were studied using an integrative taxonomic framework that combined detailed morphological examinations with multi-locus phylogenetic analyses to determine their phylogenetic affinities and taxonomic positions within the genus. Detailed descriptions of the newly proposed taxa are provided, along with comprehensive macro- and micromorphological illustrations, diagnostic characteristics, and corresponding molecular phylogenetic evidence. This study contributes to a more comprehensive understanding of the taxonomy, diversity, and evolutionary relationships of *Laccaria*.

## 2. Materials and Methods

### 2.1. Morphological Study

A total of 12 specimens utilized in this study were collected from China and Thailand. During field surveys, in situ photographs of fresh basidiomata were captured to record macromorphological features. Detailed field data, including collection localities, dates, habitats, elevations, and geographic coordinates (latitude and longitude), were documented concurrently. Color identification was performed using the Color Hexa website (http://www.colorhexa.com) (accessed on 15 December 2025) to assign codes, and morphological descriptions followed Vellinga [48]. After being thoroughly dried at 50 °C [49] in a food drier (Model SX-770; Cixi, Zhejiang, China), the specimens were stored in sealed plastic bags and deposited in the Herbarium of Cryptogams, Kunming Institute of Botany, Chinese Academy of Sciences (KUN-HKAS), the Herbarium of Mae Fah Luang University (MFLU), and Dali University. Author abbreviations and herbarium acronyms used in this study follow the Index Fungorum (http://www.indexfungorum.org) (accessed on 10 June 2026) and Index Herbariorum (https://sweetgum.nybg.org/science/ih/) (accessed on 25 June 2026), respectively. For microscopic examination, freehand sections of the basidiomata were made using a double-edged razor blade to obtain structures including the hymenium, pileipellis, and stipitipellis. Tissue sections were placed in the center of a microscope slide, mounted in 5% KOH, stained with 1% Congo red, and covered with a coverslip. Observations and photographs were taken using a Nikon ECLIPSE Ni-U compound microscope fitted with a Nikon DS-Ri2 digital camera (Nikon Corporation, Tokyo, Japan). Characteristics such as basidiospores, basidia, cystidia, hymenophoral trama, hymenium, pileipellis, and stipitipellis were recorded. Microscopic measurements were made using Image Framework v.0.9.7. For low-vacuum scanning electron microscopy (SEM), fragments of dried lamellae bearing basidiospores were mounted on aluminum stubs using double-sided conductive tape, sputter-coated with gold, and examined at 10,000× magnification with a Hitachi TM4000 PlusII microscope (Hitachi High-Tech Corporation, Tokyo, Japan) at the Dali University Analysis and Testing Center. The notation [x/y/z] specifies that measurements were made on ‘x’ basidiospores measured from ‘y’ basidiomata of ‘z’ collections. At least 50 basidiospores (measured without ornamentation) and 20 basidia (measured without including sterigmata) were measured from a single basidioma. Basidiospore dimensions are presented as (a–)b–c(–d), where ‘a’ and ‘d’ represent the minimum and maximum values of all measurements, respectively, and ‘b–c’ shows the range encompassing 95% of the measured values. ‘av.’ denotes the average value of basidiospore length × width. ‘Q’ refers to the length/width ratio of an individual basidiospore, and ‘Q_m_’ indicates the average Q value for all basidiospores ± sample standard deviation. For basidiospore shape, the value Q describes the degree of departure from a globose spore and is calculated by the length/width of the spore. In this study, basidiomata with a diameter of less than 30 mm are considered small, those between 30 and 60 mm are considered medium, and those greater than 60 mm are considered large.

### 2.2. DNA Extraction, PCR Amplification and Sequencing

DNA extraction was performed using the Ezup Column Fungi Genomic DNA Purification Kit (Sangon Biotech, Shanghai, China) according to the manufacturer’s instructions. Polymerase chain reaction (PCR) was carried out with the following primer pairs: ITS1/ITS4 [50] for the internal transcribed spacer (ITS), LR5/LR0R [51] for the large subunit ribosomal RNA gene (LSU), 5F/7cR [52] for the RNA polymerase II second largest subunit (*rpb*2), and 983F/2218R [53] for the translation elongation factor 1-α (*tef*1-α). Each PCR amplification was performed in a 25 μL reaction mixture consisting of 12.5 μL of 2 × Taq Plus Master Mix II (Vazyme Biotech Co., Ltd., Nanjing, China), 9.5 μL of double-distilled water, 1 μL of each forward and reverse primer, and 1 μL of DNA template. The amplifications were conducted on a C1000 Thermal Cycler (Bio-Rad, Beijing, China) using the following programs: for ITS and LSU, initial denaturation at 94 °C for 5 min; 35 cycles of denaturation at 94 °C for 30 s, annealing at 48 °C for 30 s, and extension at 72 °C for 90 s; followed by a final extension at 72 °C for 10 min [50]. For *rpb*2 and *tef*1-α, the program consisted of initial denaturation at 95 °C for 5 min; 35 cycles of denaturation at 95 °C for 60 s, annealing at 50 °C for 90 s, and extension at 72 °C for 60 s; with a final extension at 72 °C for 10 min. Successfully amplified PCR products were sent to Sangon Biotech (Shanghai, China) for sequencing.

### 2.3. Sequence Alignment and Phylogenetic Analysis

The newly determined nucleotide sequences of *Laccaria* species were deposited in the NCBI database (https://submit.ncbi.nlm.nih.gov/) (accessed on 14 June 2026). To verify sequence identity and screen for potential contamination, all sequences were analyzed using the Basic Local Alignment Search Tool (NCBI, https://blast.ncbi.nlm.nih.gov) (accessed on 14 June 2026), confirming their taxonomic placement within the genus *Laccaria*. Representative sequences were then selected based on a review of recent literature to assemble clusters of closely related homologs. Multiple sequence alignments were generated with MAFFT v.7 [54] and subsequently inspected and edited in BioEdit v.7 [55]. Maximum likelihood (ML) phylogenetic analysis was conducted using RAxML-HPC2 v.8.2.3 [56] on the CIPRES Science Gateway v.3.3 [57], under the GTR+G+I model of evolution with 1000 rapid bootstrap replicates for each gene partition. Given that no significant topological incongruences were detected among the individual gene trees, the four single-gene alignments were concatenated into a single dataset using Sequence Matrix-Windows-1.7.8 [58].

Bayesian phylogenetic analysis was performed using MrBayes v.3.2 [59]. The best-fit nucleotide substitution model for each partition was selected using MrModeltest v.2.3 [60]. The results showed that the best model for ITS, LSU, and *tef*1-α was GTR+I+G [Lset nst = 6, rates = invgamma, Prset statefreqpr = Dirichlet (1,1,1,1)], while the best model for *rpb*2 was HKY+I+G [Lset nst = 2, rates = invgamma, Prset statefreqpr = Dirichlet (1,1,1,1)]. Two independent runs of six Markov Chain Monte Carlo (MCMC) chains each were conducted for 10,000,000 generations, with trees sampled every 100 generations and the first 25% discarded as burn-in [61]. The runs were terminated automatically using the stoprule command once the average standard deviation of split frequencies fell below 0.01. A clade was considered strongly supported if it received bootstrap support (BS) ≥ 75% in ML analysis and a Bayesian posterior probability (PP) ≥ 0.90. The generic names of *Laccaria* and *Mythicomyces* Redhead & A.H. Sm used in this study are abbreviated as ‘*L.*’ and ‘*M.*’, respectively. The alignment was submitted to Figshare (10.6084/m9.figshare.30413050).

## 3. Results

### 3.1. Phylogenetic Analyses

**Figure 1:**
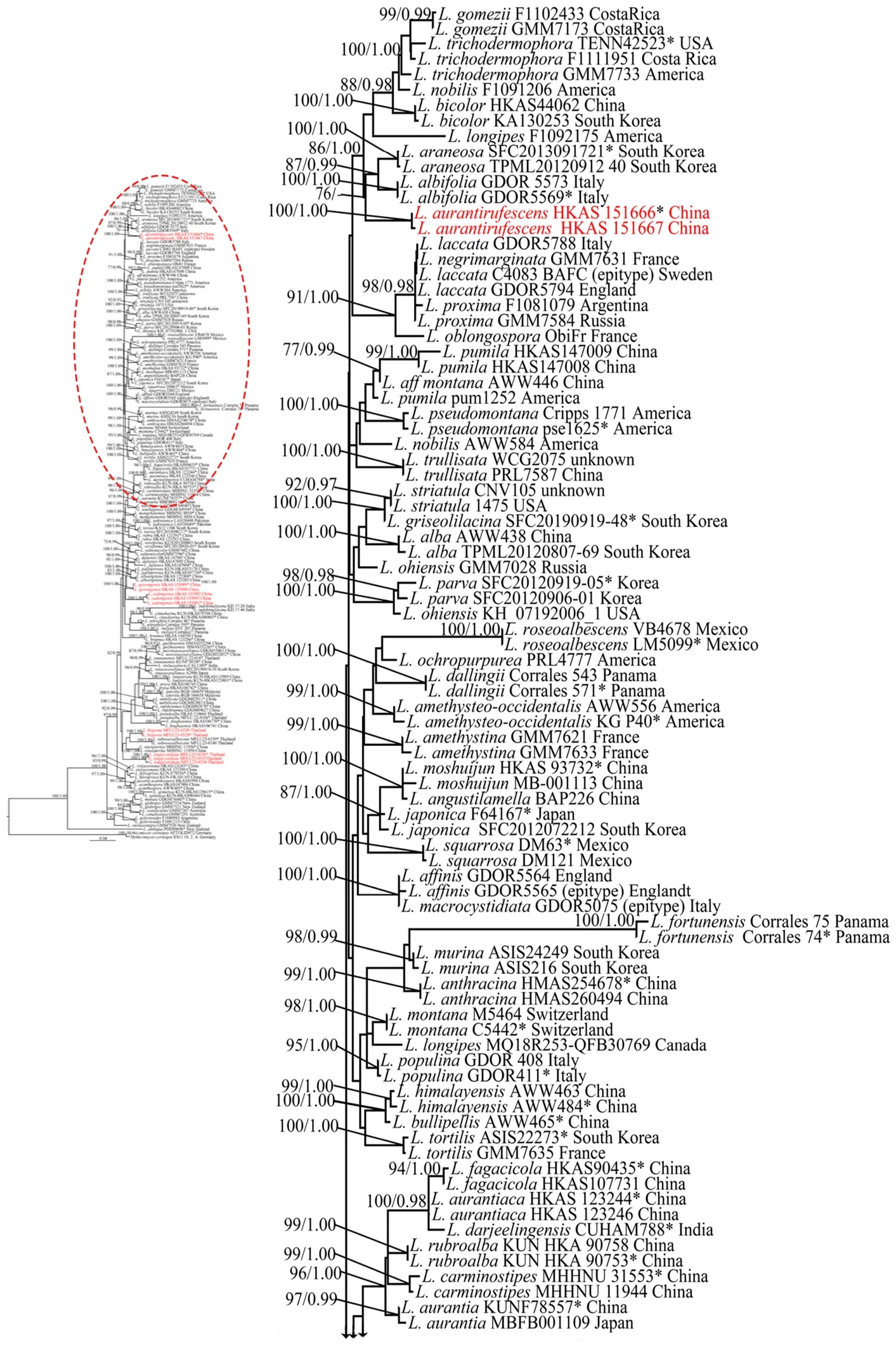

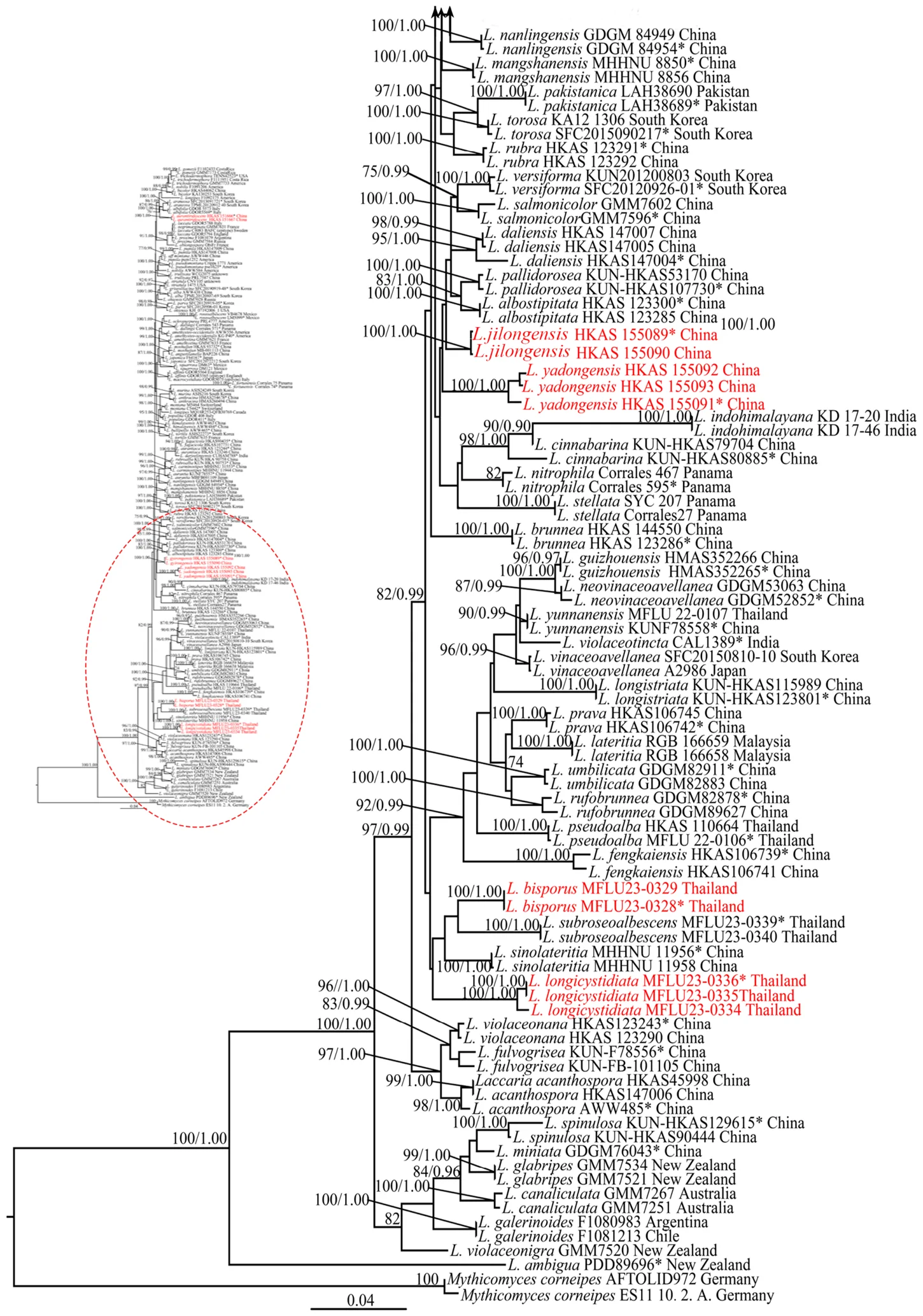
Tree results from the maximum likelihood (ML) phylogenetic tree of *Laccaria* based on the dataset of ITS +LSU + *rpb*2 + *tef*1-α. Maximum likelihood bootstrap proportions equal to or higher than 75% and Bayesian posterior probabilities equal to or higher than 0.90 are indicated at nodes. New species are indicated in red. Holotype specimens are marked with an asterisk (*).

Multi-gene phylogenetic analysis showed that 12 specimens of *Laccaria* collected from China and Thailand formed five distinct and well-supported clades (Figure 1). Combined with morphological comparisons, these clades were identified as five new species, namely *L. aurantirufescens*, *L. bisporus*, *L. jilongensis*, *L. longicystidiata*, and *L. yadongensis*.

The phylogenetic positions of the new species are as follows: *L. aurantirufescens* forms a sister clade with *L. araneosa* and *L. albifolia*; *L. bisporus* is closely related to *L. subroseoalbescens*; *L. longicystidiata* clusters with *L. sinolateritia*; and both *L. jilongensis* and *L. yadongensis* represent well-supported independent lineages (Figure 1).

The phylogenetic analyses were based on a combined dataset of ITS, LSU, *rpb*2, and *tef*1-*α* sequences, comprising 174 specimens with 3979 characteristics (ITS: 1–660 bp, LSU: 661–1608 bp, *rpb*2: 1609–2730 bp, *tef*1-α: 2731–3979 bp) (Table 1), with *Mythicomyces corneipes* Redhead & A.H. Sm selected as the outgroup [18,23,26,41,42]. RAxML and Bayesian analyses yielded largely congruent topologies, with bootstrap support (BS) ≥ 75% and Bayesian posterior probabilities (PP) ≥ 0.90 considered strong support. The best RAxML tree had a final likelihood value of −26,116.324664. The matrix contained 1988 distinct alignment patterns, with 55.33% undetermined characteristics or gaps. Estimated base frequencies were as follows: A = 0.257256, C = 0.215983, G = 0.250745, T = 0.276016; substitution rates AC = 1.188044, AG = 5.315002, AT = 1.531121, CG = 1.015266, CT = 7.737532, and GT = 1.000000; the gamma distribution shape parameter was α = 0.270808.

### 3.2. Taxonomy

***Laccaria aurantirufescens*** G. Zhao & S.M. Tang, sp. nov.

**Figure 2:**
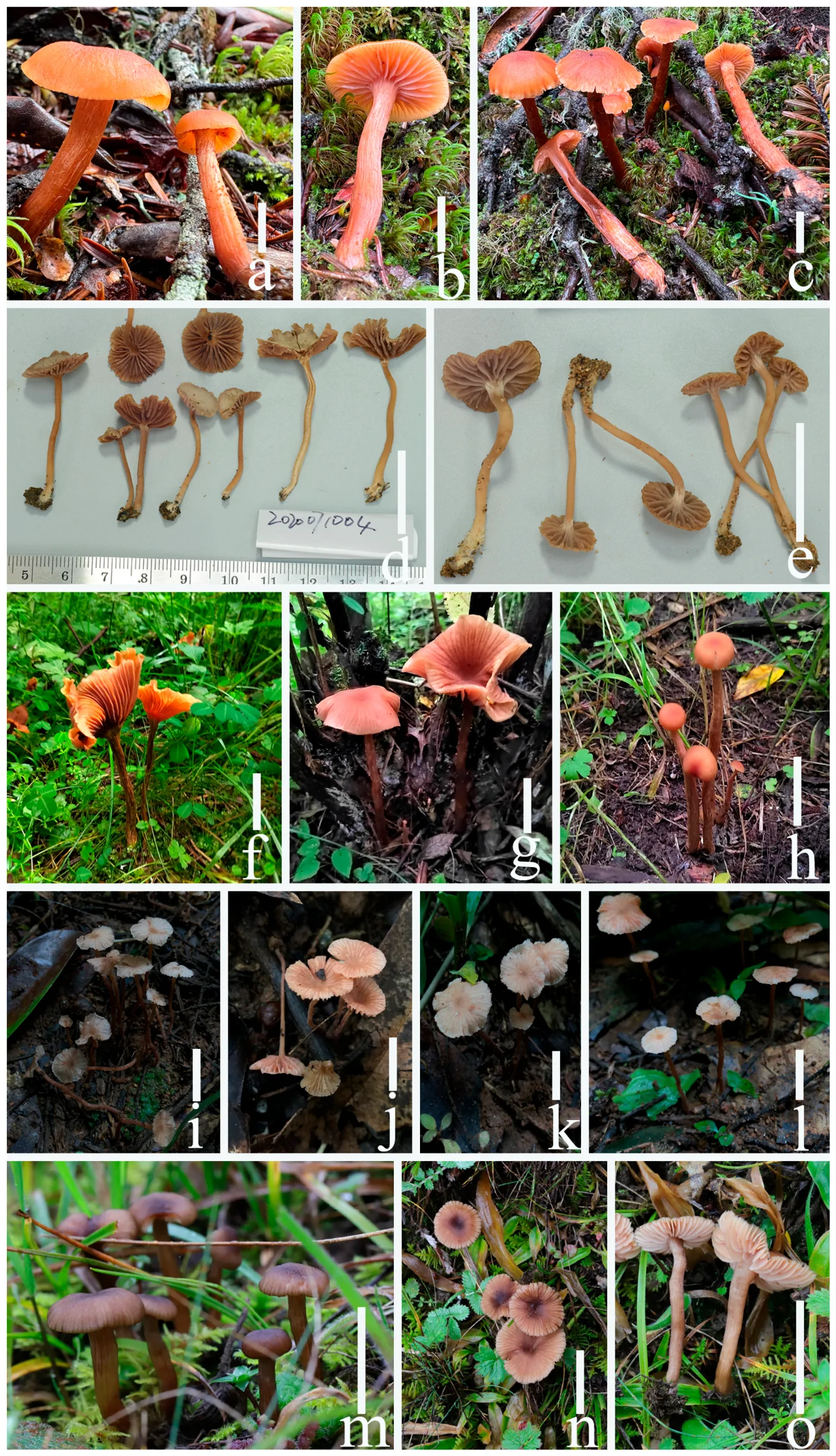
Basidiomata of *Laccaria*. (**a**–**c**) *L. aurantirufescens*; (**d**–**e**) *L. bisporus*; (**f**–**h**) *L. jilongensis*; (**i**–**l**) *L. longicystidiata*; (**m**–**o**) *L. yadongensis*. Scale bars: (**a**–**o**) = 2 cm.

**Notes**. From a morphological perspective, *L. aurantirufescens*, *L. cinnabarina* J. Li and Y. Y. Cui, and *L. nanlingensis* M. Zhang have orange-red pileus, sinuate lamellae, making them prone to confusion, and are longitudinally striate on the stipes. However, *L. cinnabarina* has a larger pileus (up to 9 cm), larger basidiospores (av. 8.3 × 8.0 μm vs. 7.7 × 7.5 μm) and absent cystidia [62]. *Laccaria nanlingensis* has a relatively large pileus (30–55 mm), smaller basidiospores (av. 7.0 × 6.4 μm vs. 7.7 × 7.5 μm), and shorter, sparser spines on the basidiospores (0.5–1 μm vs. 1–2.5 μm), absent pleurocystidia, present caulocystidia, and cheilocystidia are longer (40–60 µm vs. 18–34 µm) [18].

**Figure 3:**
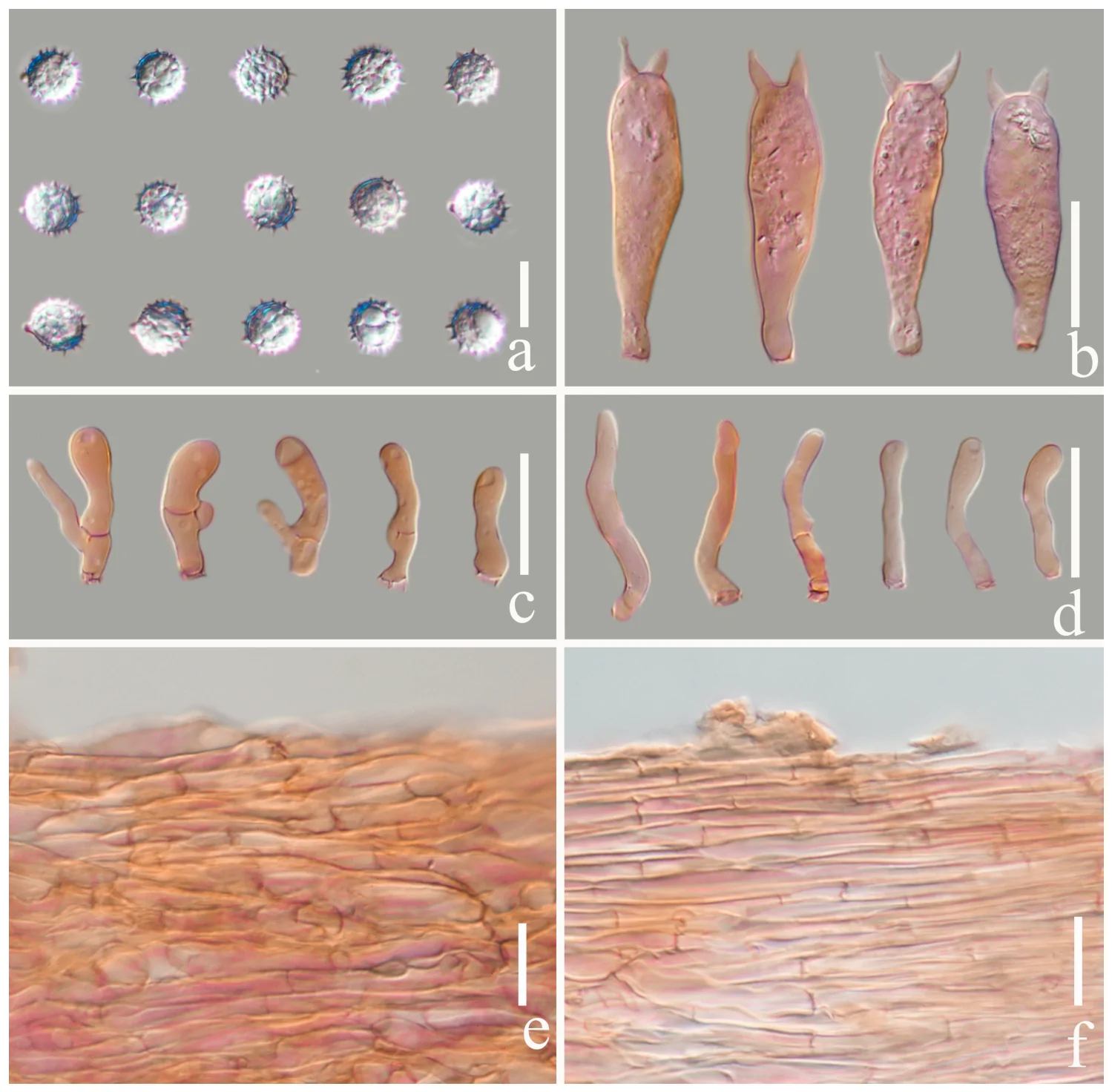
***Laccaria aurantirufescens*** (holotype, KUN-HKAS 151666). (**a**) Basidiospores; (**b**) basidia; (**c**) pleurocystidia; (**d**) cheilocystidia; (**e**) pileipellis; (**f**) stipitipellis. Scale bars: (**a**) = 10 μm; (**b**–**f**) = 20 μm.

Figure 2a–c, Figure 3 and Figure 8a

**Chinese Name**: 橙赭蜡蘑 (Cheng Zhe La Mo)

**Fungal Names number:** FN 573592

**Diagnosis**. *Laccaria aurantirufescens* is characterized by medium-small basidiomata, orange-red to russet pileus, lamellae sparse, stipe yellowish-brown to reddish-brown, basidiospores globose to subglobose, and basidia that are mostly four-spored, rarely two-spored. Phylogenetically, this species forms a sister clade with *L. araneosa* H.J. Cho & Y.W. Li (TPML20120912-40, holotype) and *L. albifolia* Dovana & Para (GDOR5569, holotype), with ITS sequence divergences of 2.01% (12/596) and 2.16% (13/602), respectively.

**Etymology**. ‘*aurantirufescens*’ refers to the orange-red to russet color of the pileus.

**Holotype**. China, Yunnan Province, Dali Bai Autonomous Prefecture, JianChuan County, Laojunshan Mountain, 26°09′06.42″ N, 100°13′46.15″ E, elev. 2382 m, 6 July 2025, G. Zhao & S. M. Tang, 20250706-17 (KUN-HKAS 151666).

**Description**. ***Pileus*** 8–35 mm in diam., hemispherical, applanate to plano-convex, orange-red (#ff7f24) to russet (#a0522d), with striations on the surface, slightly depressed to depressed at the center, smooth, shiny, margin thin, undulate; context 1–3 mm. ***Lamellae*** sparse, ventricose, sinuate to adnate with decurrent tooth, concolorous with pileus, 1–2 mm wide; lamella edge even or entire, sometimes wavy; lamellulae in 2–4 tiers. ***Stipe*** 15–80 × 2–6 mm, cylindrical, central or eccentric, equal in width or enlarged at the base and tapering upwards, yellowish-brown (#cc9966) to reddish-brown (#8b4513), with longitudinal fibrous striations on the surface. ***Odor*** and taste not observed.

***Basidia*** 34–47 × 9–15 μm, av. 41.4 ± 2.9 × 11.7 ± 1.1 μm, clavate, mostly four-spored, rarely two-spored; sterigmata 3–9 μm long. ***Basidiospores*** (excluding ornamentation) [120/2/2]6.3–9.4 × 6.0–9.1 μm, av. 7.7 ± 0.5 × 7.5 ± 0.5 μm, Q_m_ = 1.0–1.1, Q_av._ = 1.02 ± 0.01, subglobose to globose, echinulate, spines 1–2.5 µm long, 1–2 µm wide at the base, crowded. ***Cheilocystidia*** 18–34 ×2–6 μm, av. 24.9 ± 3.5 × 4.0 ± 0.8 μm, narrowly clavate, flexuose, thin-walled, colorless and hyaline, abundant. ***Pleurocystidia*** 13–24 × 2–6 μm, av. 18.8 ± 2.6 × 4.1 ± 0.7 μm, subclavate, narrowly clavate, occasionally branched, flexuose, thin-walled, hyaline. ***Lamellar trama*** 60–120 μm thick, regular, composed of slightly thick-walled, filamentous hyphae 2–8 μm wide. ***Lamellar edge*** with abundant sterile basidia, composed of clavate, cylindrical inflated cells 8–20 × 6–14 μm, thin-walled, colorless, similar to basidioles in shape. ***Subhymenium*** 10–16 μm thick, tightly interwoven, composed of fusiform to irregular cells, 3–8 × 2–6 μm, av. 6.4 ± 0.9 × 4.3 ± 0.8 μm. ***Pileipellis*** 60–110 μm thick, colorless in 5% KOH solution, composed of appressed, parallel, simply septate, thin-walled, cylindrical, filamentous hyphae 4–12 μm wide. ***Stipitipellis*** composed of appressed, parallel, simply septate, thick-walled hyphae 2–10 μm wide; stipe trama composed of longitudinally arranged, colorless in 5% KOH solution, clavate terminal cells, infrequently branching, septate, thick-walled, hyaline hyphae 3–10 μm wide. ***Caulocystidia*** absent. ***Clamp connections*** present at some septa in pileipellis, lamellae, and stipitipellis.

**Habitat and phenology**. Growing scattered and gregarious in mixed coniferous and broad-leaved forests.

**Additional specimens examined**. China, Yunnan Province, Dali Bai Autonomous Prefecture, Jianchuan County, Laojun Mountain, 26°09′06.42″ N, 100°13′46.15″ E, elev. 2382 m, 6 July 2025, G. Zhao & S. M. Tang, paratype, 20250706-35 (KUN-HKAS 151667).

According to the phylogenetic analysis (Figure 1), *L. aurantirufescens* forms a sister clade with *L. araneosa* and *L. albifolia*. However, *L. araneosa* lacks cystidia and has orange-brown to light brown pileus, larger basidia (42–52 × 11–14 μm vs. 34–47 × 9–15 μm) and basidiospores (8.0–9.0 × 7.5–9.0 μm vs. 6.3–9.4 × 6.0–9.1 μm) [43]. *Laccaria albifolia* has an orange pileus with a whitish margin, shorter spore spines (≤1.2 μm) with a narrower base (<1 μm), and cheilocystidia filamentous to irregularly flexuose (17–80 × 2–4 μm) [20]. Sequence divergence analysis shows that the ITS sequence divergence between *L. aurantirufescens* (KUN-HKAS 151666, holotype) and *L. araneosa* (TPML20120912-40, holotype) is 2.01% (12/596), and that between *L. aurantirufescens* and *L. albifolia* (GDOR5569, holotype) is 2.16% (13/602).

***Laccaria bisporus*** G. Zhao & S.M. Tang, sp. nov.

**Figure 4:**
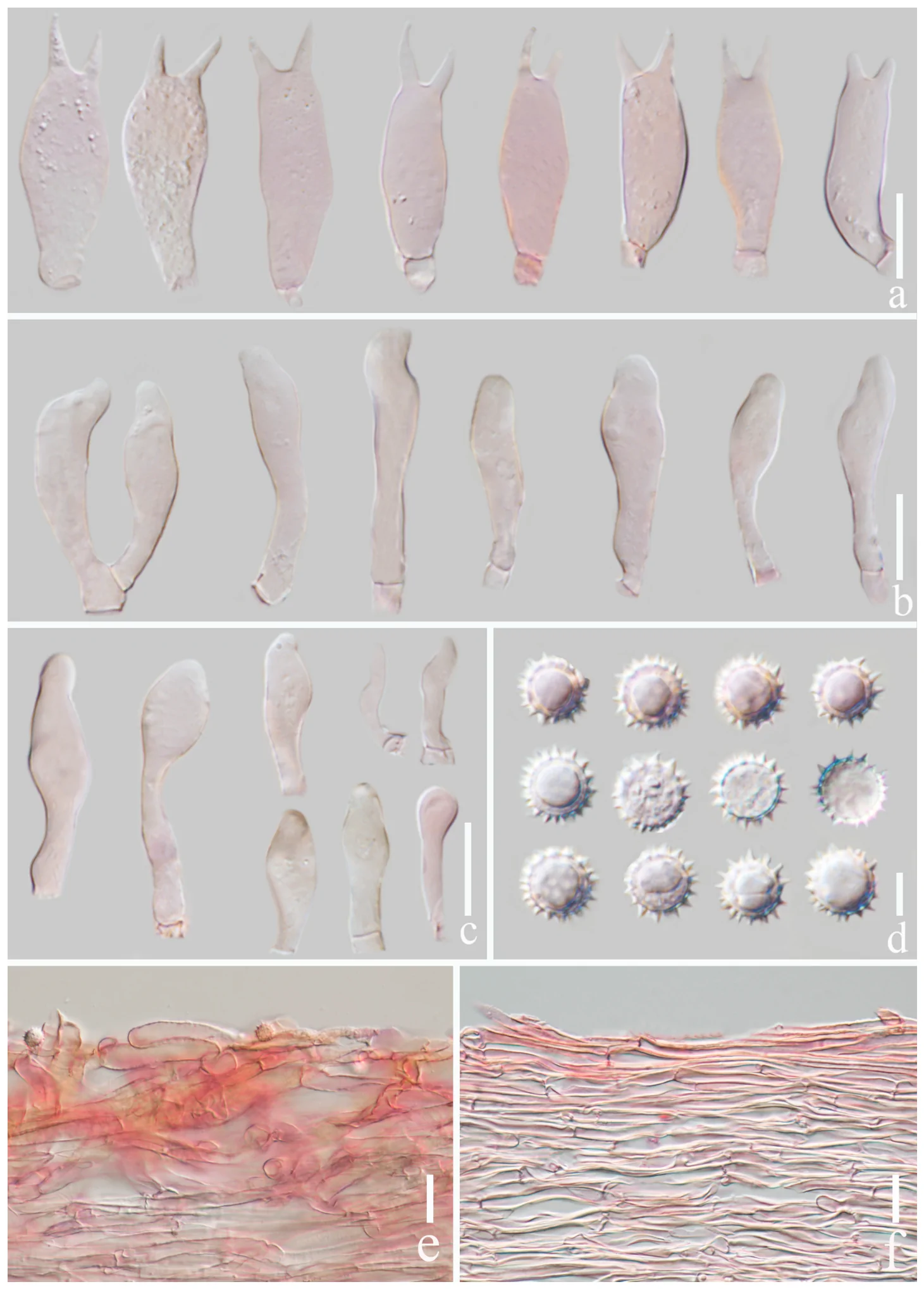
***Laccaria bisporus*** (holotype, MFLU23-0328 & KUN-HKAS 151668). (**a**) Basidia; (**b**) cheilocystidia; (**c**) pleurocystidia; (**d**) basidiospores; (**e**) pileipellis; (**f**) stipitipellis. Scale bars: (**a**–**c**) = 20 μm; (**d**) = 10 μm; (**e**,**f**) = 20 μm.

Figure 2d–e, Figure 4 and Figure 8b

**Chinese Name**: 双孢蜡蘑 (Shuang Bao La Mo)

**Fungal Names number:** FN 573594

**Diagnosis.** *Laccaria bisporus* is characterized by small basidiomata, pale orange to orange-white pileus, distant lamellae, brown to yellowish-brown stipe, basidia that are 2-spored, 4-spored not observed. Phylogenetically, this species differs from the closely related species *L. subroseoalbescens* S.M. Tang & S.H. Li. (MFLU23-0339, holotype) by 5.75% (37/644) in ITS sequence divergence.

**Etymology.** The epithet ‘*bisporus*’ reflects the two basidiospores on the basidia.

**Holotype**. Thailand, Chiang Mai Province, Pa Pae Taeng District, Mushroom Research Center, elev. 576 m, 11 August 2020, S. M. Tang, 2020081160 (MFLU23-0328).

**Description. *Pileus*** 7–17 mm in diam., applanate when seen from aside, pale orange (#fad6a5), orange white (#fef9e7), when dry moisture loss of moisture or with age becoming whitish, clearly striate on the surface; with a small papilla when young, becoming depressed to subumbilicate with age; margin reflexed; context less than 1 mm, pale orange (#fad6a5). ***Lamellae*** distant, arcuate, adnate with decurrent tooth, orange white (#fef9e7) when young, becoming pale orange (#fad6a5) with age, 1–2 mm wide; lamella edge even or entire, occasionally undulate; lamellulae in 3–4 tiers. ***Stipe*** 27–36 × 0.8–2.4 mm, cylindrical, central, brown (#964b00) to yellowish brown (#cc7722), smooth, basal mycelium white (#ffffff); stipe context fistulose, yellowish brown (#cc7722). ***Odor*** and taste not observed.

***Basidia*** 25–34 × 10–14 μm, av. 30 ± 3.2 × 12 ± 1.1 μm, clavate, consistently 2-spored (4-spored not observed), sterigmata 6–10 μm long. ***Basidiospores*** (excluding ornamentation) [108/2/4] 8.1–10.6 × 7.2–10.1 μm, av. 9.2 ± 0.7 × 8.8 ± 0.7 μm, Q_m_ = 1.00–1.22, Q_av._ = 1.04 ± 0.08, globose to subglobose, hyaline, echinulate, spines 1–3 μm long, 0.5–1.5 μm wide at the base, crowded. ***Cheilocystidia*** 22–42 × 5–7 μm, av. 32 ± 8.3 × 6.5 ± 0.8 μm, clavate to narrowly clavate, rarely flexuose, thin-walled, colorless and hyaline, abundant. ***Pleurocystidia*** 11–19 × 3–6 μm, av. 16 ± 2.6 × 4 ± 1.1 μm, narrowly clavate to subclavate, rarely flexuose, thin-walled, hyaline, abundant. ***Lamellar trama*** 60–70 μm thick, regular, composed of slightly thick-walled, filamentous hyphae 3–7 μm wide. ***Lamellar edge*** with abundant sterile basidia, composed of clavate, cylindrical inflated cells 6–12 × 4–10 μm, thin-walled, colorless, similar to basidioles in shape. ***Subhymenium*** 13–16 μm thick, tightly interwoven, composed of fusiform to irregular cells, 3–7 × 2–4 μm, av. 6 ± 1.1 × 3.2 ± 0.3 μm. ***Pileipellis*** 100–127 μm thick, appearing pale pink in 5% KOH solution, composed of appressed, parallel, simply septate, thin-walled, cylindrical, filamentous hyphae 4–10 μm wide. ***Stipitipellis*** composed of appressed, parallel, simply septate, thick-walled, hyphae 6–8 μm wide; stipe trama composed of longitudinally arranged, septate, thick-walled hyphae 6–10 μm wide, with clavate terminal cells, infrequently branching, appearing colorless in 5% KOH solution. ***Caulocystidia*** 29–45 × 3–7 μm, av. 38.5 ± 4.6 × 4.5 ± 0.9 μm, subclavate to narrowly clavate, flexuose, thin-walled, hyaline. Clamp connections present at some septa in pileipellis, lamellae, and stipitipellis.

**Habitat and phenology.** Scattered, gregarious or caespitose on ground in *Hopea*.

**Additional specimens examined.** Thailand, Chiang Mai Province, Pa Pae Taeng District, Mushroom Research Center, 13 August 2020, elev. 587 m, S. M. Tang, 2020081161 (MFLU23-0329 & KUN-HKAS 151669).

**Notes**: Morphologically, *L. bisporus* is easily confused with *L. fengkaiensis* F. Li and *L. pseudoalba* S.M. Tang & S.H. Li due to their shared orange-toned pileus. However, *L. fengkaiensis* has a larger pileus (7–17 mm vs. 50–90 mm), wider lamellae (1–2 mm vs. up to 8 mm), 4-spored and narrower basidia (10–14 μm vs. 6–8.5 μm), and smaller basidiospores (av. 9.2 × 8.8 μm vs. 5.8 × 5.7 μm) with shorter spines (1–3 μm vs. 0.5–1.2 μm) [44]. *Laccaria pseudoalba* has a thinner pileipellis (100–127 μm vs. 70–100 μm) and wider pleurocystidia (15–31 × 6–8 μm vs. 11–19 × 3–6 μm) [27].

Multi-locus phylogenetic analysis (Figure 1) reveals that *L. bisporus* is closely related to *L. subroseoalbescens*. However, *L. subroseoalbescens* is distinguished by its smaller pileus (2–8 mm vs. 7–17 mm), basidia predominantly 4-spored (rarely 2-spored, vs. strictly 2-spored), smaller basidiospores (av. 8.3 × 7.8 μm vs. 9.2 × 8.8 μm), and larger pleurocystidia (36–59 × 5–8 μm vs. 11–19 × 3–6 μm). In addition, the ITS sequence divergence between *L. bisporus* (MFLU23-0328, holotype) and *L. subroseoalbescens* (MFLU23-0339, holotype) is 5.75% (37/644), further supporting its status as a distinct species [27].

***Laccaria jilongensis*** G. Zhao & S.M. Tang, sp. nov.

**Figure 5:**
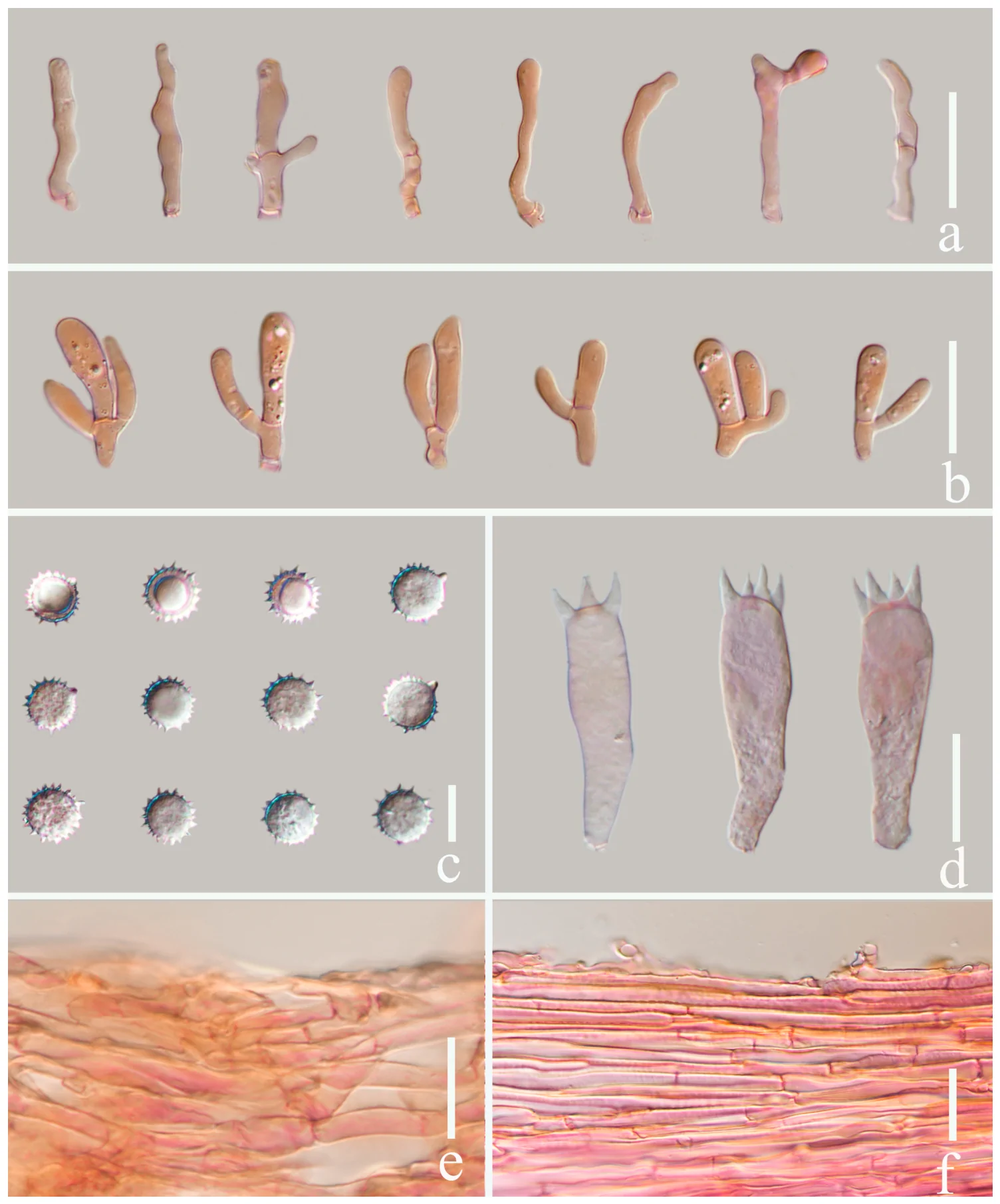
***Laccaria jilongensis*** (KUN-HKAS 155089, holotype). (**a**) Cheilocystidia; (**b**) pleurocystidia; (**c**) basidiospores; (**d**) basidia; (**e**) pileipellis; (**f**) stipitipellis. Scale bars: (**a**,**b**) = 20 μm; (**c**) = 10 μm; (**d**–**f**) = 20 μm.

Figure 2f–h, Figure 5 and Figure 8c

**Chinese Name**: 吉隆蜡蘑 (Ji Long La Mo)

**Fungal Names number:** FN 573932

**Diagnosis.** *Laccaria jilongensis* is characterized by large basidiomata, pileus up to 11 cm in diameter, orange-red, lamellae sparse, stipe up to 15 cm long, basidiospores globose to subglobose, basidia clavate, and cystidia present. Phylogenetically, this species forms a sister clade with *L. albostipitata* S.M. Tang, K.D. Hyde (HKAS 123300), with an ITS sequence divergence of 3.49% (20/572).

**Etymology.** The epithet ‘*jilongensis*’ refers to the collection locality of the specimen: Jilong County, Xigaze City, Xizang Autonomous Region.

**Holotype**. China, Xizang Autonomous Region, Xigaze City, Jilong County, 28°39′12.62″ N, 85°35′40.20″ E, elev. 2744 m, 28 July 2025. G. Zhao & S. M. Tang, 20250728-75 (KUN-HKAS 155089).

**Description. *Basidiomata*** are relatively large. ***Pileus*** 5–110 mm in diam., hemispherical when young, becoming plano-convex to infundibuliform with age, centrally depressed, margin involute, orange-red (#ff7f24), with the color gradually fading from the center to the edge, when losing moisture or with age becoming orange-brown (#b16002), distinctly striate on the surface, smooth, shiny, margin very thin, crenulate to eroded; pileus context thin, below 2–4 mm thick. ***Lamellae*** distant, adnate with decurrent tooth, orange-brownish-red, 1–2 mm wide; lamella edge even or entire, sometimes undulate; lamellulae in 1–3 tiers. ***Stipe*** 50–150 × 2–8 mm, cylindrical, central, fistulose, equal in width and enlarged at the base, nearly subclavate, reddish brown (#a52a2a), surface with longitudinal fibrous striations and fine small scales. ***Odor*** and taste not observed.

***Basidia*** 31–44 × 9–16 μm, av. 37.2 ± 2.9 × 12.8 ± 1.9 μm, clavate, mostly 4-spored, rarely 2-spored; sterigmata 2–9 μm long. ***Basidiospores*** (excluding ornamentation) [100/2/2] 7.6–9.9 × 7.3–9.7 μm, av. 8.8 ± 0.6 × 8.7 ± 0.7 μm, Q_m_ = 1.0–1.1, Q_av._ = 1.01 ± 0.02, subglobose to globose, hyaline, echinulate, spines 2–4 μm long, 1–2 μm wide at the base, crowded. ***Cheilocystidia*** 22–33 × 2–6 μm, av. 28.1 ± 2.8 × 4.0 ± 0.7 μm, narrowly clavate, occasionally branched, flexuose, thin-walled, colorless and hyaline, abundant. ***Pleurocystidia*** 13–22 × 3–5 μm, av. 17.3 ± 2.1 × 4.1 ± 0.6 μm, subclavate, narrowly clavate, branched, thin-walled, hyaline. ***Lamellar trama*** 60–120 μm thick, regular, composed of slightly thick-walled, filamentous hyphae 2–8 μm wide. ***Lamellar edge*** with a greater number of sterile basidia, composed of clavate, cylindrical inflated cells 6–18 × 4–12 μm, thin-walled, colorless, similar in shape to basidioles. ***Subhymenium*** 10–14 μm thick, tightly interwoven, composed of fusiform or irregular cells, 2–8 × 2–6 μm. ***Pileipellis*** 60–120 μm thick, colorless and hyaline in 5% KOH, composed of appressed, parallel, simply septate, thin-walled, cylindrical filamentous hyphae 4–12 μm wide, clamped, mostly hyaline but some with brown pigmentation. ***Stipitipellis*** composed of appressed, parallel, simply septate, thin-walled hyphae 2–8 μm wide; stipe trama composed of longitudinally arranged cells, colorless in 5% KOH solution, with clavate terminal cells, infrequently branching, septate, thick-walled, hyaline hyphae 3–8 μm wide. ***Caulocystidia*** absent. ***Clamp connections*** present at some septa in pileipellis, lamellae, and stipitipellis.

**Habitat and phenology.** Growing scattered to small groups on moist humus soil in mixed coniferous and broad-leaved forests.

**Additional specimens examined.** China, Xizang Autonomous Region, Linzhi City, 29°04′29.33″ N, 94°24′43.01″ E, elev. 3150 m, 18 July 2025, G. Zhao & S. M. Tang, 20250718-49 (KUN-HKAS 155090)

**Notes**: Morphologically, *L. jilongensis* is easily confused with *L. nanlingensis* M. Zang. and *L. cinnabarina* J. Li and Y. Y. Cui. due to their shared orange-red pileus. However, *L. nanlingensis* has smaller basidiomata (pileus 30–55 mm vs. 5–110 mm), smaller basidiospores (6.5–7.5 × 6–7 μm vs. 7.6–9.9 × 7.3–9.7 μm), shorter spines (0.5–1 μm vs. 2–4 μm), and lacks pleurocystidia but presents caulocystidia [18]. *Laccaria. cinnabarina* has smaller basidiomata (pileus diameter 1–9 cm vs. 5–11 cm), dark brown center and orange-red margin, completely lacks cystidia, has shorter spines (2 μm vs. 2–4 μm), and lacks brown pigmentation in the pileipellis [62].

Multi-locus phylogenetic analysis (Figure 1) reveals that *L. jilongensis* is closely related to *L. albostipitata*. However, *L. albostipitata* can be readily distinguished by its comparatively diminutive basidiomata (pileus 18–24 mm vs. 5–110 mm; stipe 34–49 mm vs. 50–150 mm), pileus ranging from soft orange to whitish, and notably shorter basidiospore spines (1–2 μm vs. 2–4 μm in length). The ITS sequence divergence between *L. jilongensis* (HKAS 155089) and *L. albostipitata* (HKAS 123300) is 3.49% (20/572) [28].

***Laccaria longicystidiata*** G. Zhao & S.M. Tang, sp. nov.

**Figure 6:**
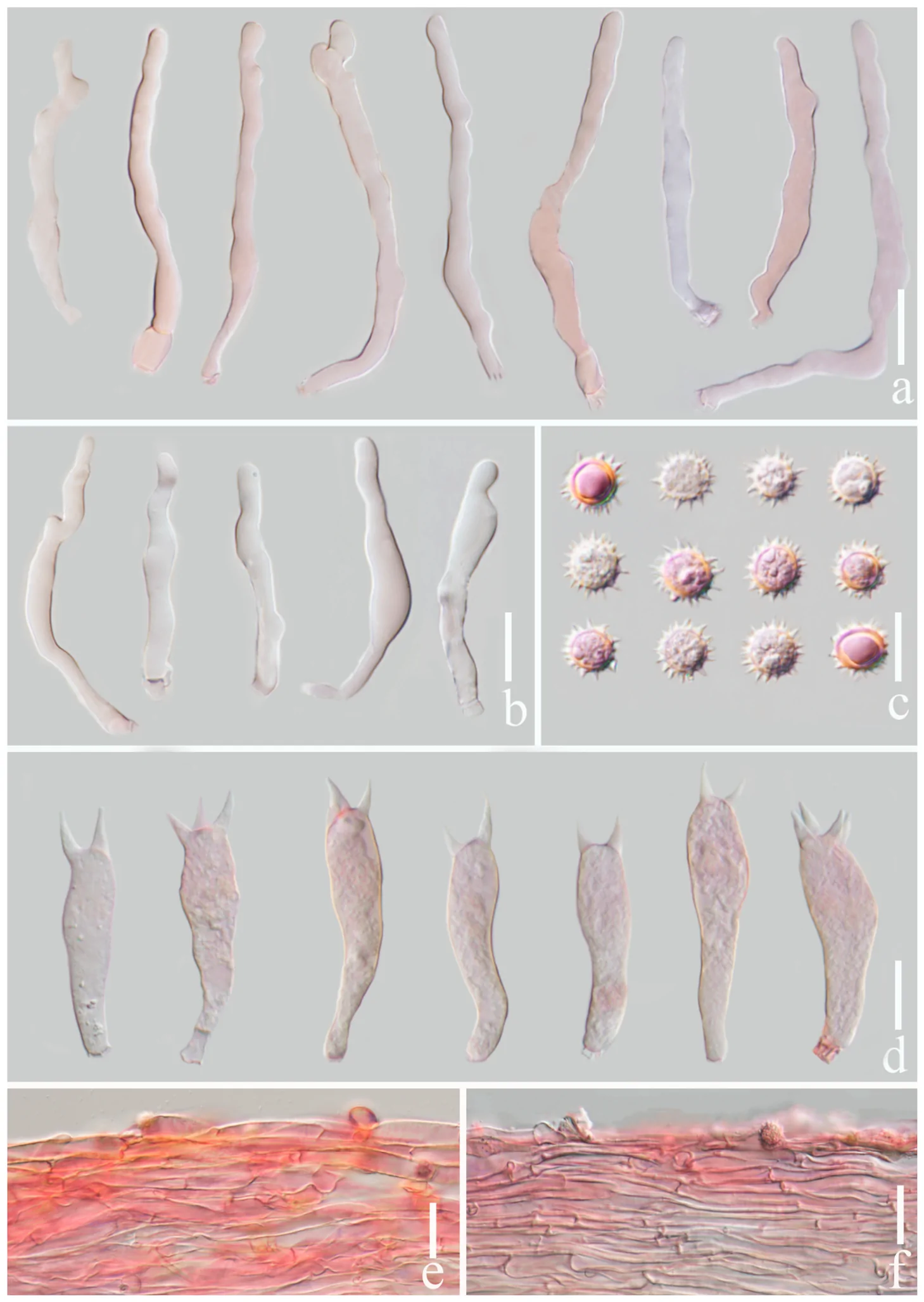
***Laccaria longicystidiata*** (holotype, MFLU23-0336 & KUN-HKAS 151672). (**a**) Cheilocystidia; (**b**) pleurocystidia; (**c**) basidiospores; (**d**) basidia; (**e**) pileipellis; (**f**) stipitipellis. Scale bars: (**a**–**d**) = 10 μm; (**e**,**f**) = 20 μm.

**Notes**. From morphology, *L. longicystidiata* is prone to confusion with *L. subroseoalbescens* S.M. Tang & S.H. Li, *L. fengkaiensis* F. Li, and *L. umbilicate* M. Zhang, as all four species share orange-toned pileus. However, *L. subroseoalbescens*, has a larger pileus (9–20 mm vs. 2–8 mm) and light brown to brown lamellae and stipe, shorter stipe (5–13 mm vs. 18–36 mm), shorter cheilocystidia (23–37 × 4–8 μm vs. 57–122 × 4–9 μm), completely lacks pleurocystidia and caulocystidia, and has a thinner trama (74–90 μm vs. 197–254 μm) of narrower hyphae (2–5 μm vs. 8–10 μm wide) [27]. *Laccaria fengkaiensis* has larger pileus (pileus diameter 50–90 mm vs. 9–20 mm in diam.), broad lamellae (up to 8 mm vs. 1–3 mm) that are pastel red to grayish red, and a robust stipe (60–100 × 6–9 mm vs. 27–36 × 1–2 mm) concolorous with the pileus, smaller basidiospores (av. 5.8 × 5.7 μm vs. av. 7.8 × 7.3 μm) and shorter spines (0.5–1.2 μm vs. 2–3 μm) [44]. *Laccaria umbilicate* lacks pleurocystidia, possesses longer, narrower, slightly thick-walled caulocystidia (28–38 × 4–6 μm), has larger basidiospores (av. 9.0 × 8.7 μm vs. 7.8 × 7.3 μm) with subdistant spines, and narrower tramal hyphae (3–6 μm wide) [18].

Figure 2i–l, Figure 6 and Figure 8d

**Chinese Name**: 长囊体蜡蘑 (Chang Nang Ti La Mo) 

**Fungal Names number:** FN 573593

**Diagnosis.** *Laccaria longicystidiata* is characterized by small basidiomata, pale orange to grayish-orange pileus, lamellae distant, stipe light brown to brown, and larger cheilocystidia. Phylogenetically, this species differs from the allied species *L. sinolateritia* Y.D. Xu & Z.M. He (MHHNU 11956, holotype) by 4.77% (28/587) in ITS sequence divergence.

**Etymology.** The epithet ‘*longicystidiata*’ refers to its having long cheilocystidia.

**Holotype.** Thailand, Chiang Rai Province, Mae Sai District, Ko Chang, elev. 678 m, 10 July 2020, S. M. Tang, 2020071004 (MFLU23-0336).

**Description. *Pileus*** 9–20 mm in diam., plano-concave, convex to applanate, pale orange (#fad6a5), grayish orange (#c4a484), when dry moisture loss of moisture or with age becoming whitish, smooth on the surface; slightly depressed to depressed at the centre; margin straight; context 0.7–1 mm, light brown (#b5651d) to brown (#964b00). ***Lamellae*** distant, sinuate to adnate with decurrent tooth, light brown (#b5651d) to brown (#964b00), 1–3 mm wide; lamella edge even or entire, sometimes undulate; lamellulae in 2–3 tiers. ***Stipe*** 18–36 × 1–2 mm, cylindrical, central, equal with an enlarged base, rarely subclavate, light brown (#b5651d) to brown (#964b00), fistulose, smooth, basal mycelium white (#ffffff). ***Odor*** and taste not observed.

***Basidia*** 45–55 × 10–13 μm, av. 50 ± 4.2 × 12 ± 1.2 μm, clavate, mostly 4-spored, rarely 2-spored, sterigmata 6–10 μm long. ***Basidiospores*** (excluding ornamentation) [150/3/3] 6.3–9.3 × 6.3–8.9 μm, av. 7.8 ± 0.8 × 7.3 ± 0.7 μm, Q = 1.00–1.23, Q_m_ = 1.08 ± 0.09, globose to subglobose, echinulate, spines 2–3 μm long, 1–2 μm wide at the base, crowded. ***Cheilocystidia*** 57–122 × 4–9 μm, av. 87 ± 20.5 × 6 ± 1.1 μm, narrowly clavate, thin-walled, colorless and hyaline, abundant. ***Pleurocystidia*** 25–65 × 6–8 μm, av. 51 ± 19.0 × 7 ± 1.1 μm, narrowly clavate to subclavate, flexuose or mucronate, thin-walled, hyaline, abundant. ***Lamellar trama*** 197–254 μm thick, regular, composed of slightly thick-walled, filamentous hyphae 8–10 μm wide. ***Subhymenium*** 21–29 μm thick, tightly interwoven, composed of fusiform or irregular cells, 6–9 × 4–6 μm, av. 8.0 ± 1.1 × 4.7 ± 0.7 μm. ***Pileipellis*** 90–125 μm thick, hyaline in 5% KOH solution, composed of appressed, parallel, simply septate, thin-walled, cylindrical, filamentous hyphae 6–10 μm wide. ***Stipitipellis*** composed of appressed, parallel, simply septate, thick-walled hyphae 5–9 μm wide; stipe trama composed of longitudinally arranged, septate, thick-walled hyphae 7–12 μm wide; hyphae with clavate terminal cells, infrequently branching, hyaline in 5% KOH solution. ***Caulocystidia*** 10–15 × 7–8 μm, narrowly clavate to subclavate. ***Clamp connections*** present on some septa in pileipellis, lamellae, and stipitipellis.

**Habitat and phenology**. Scattered, gregarious or caespitose on ground in *Fagus* and *Dipterocarpus*.

**Additional specimens examined**. Thailand, Chiang Rai Province, Mae Sai district, Ko Chang, elev. 608 m, 10 July 2020, S. M. Tang, 2020071002 (MFLU23-0335 & KUN-HKAS 151671); ibid, elev. 728 m, 10 July 2020, S. M. Tang, 2020071001 (MFLU23-0334 & KUN-HKAS 151670).

Multi-locus phylogenetic analysis (Figure 1) indicates that *L. longicystidiata* and *L. sinolateritia* are closely related. However, *L. sinolateritia* lacks cheilocystidia and pleurocystidia, but presents abundant pileocystidia and larger basidiospores (7.5–10.5 × 7.5–10.5 μm vs. 6.3–9.3 × 6.3–8.9 μm), a tomentose and striate pileus, broader lamellae (approximately 5 mm), and a stipe surface covered with white fibrils. The ITS sequence divergence between *L. longicystidiata* (MFLU23-0336, holotype) and *L. sinolateritia* (MHHNU 11956, holotype) is 4.77% (28/587), further supporting their status as independent species [19].

***Laccaria yadongensis*** G. Zhao & S.M. Tang, sp. nov.

**Figure 7:**
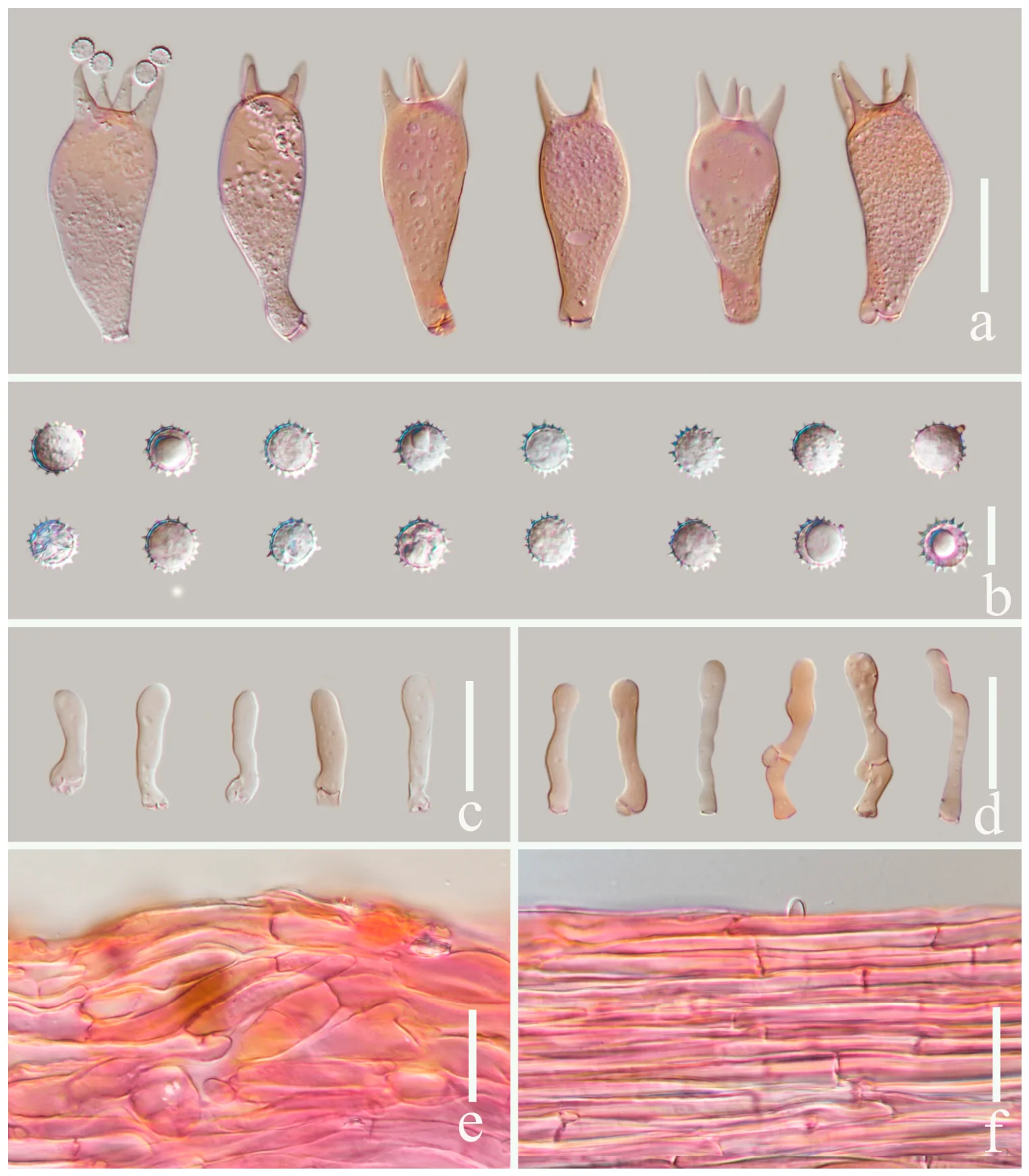
***Laccaria yadongensis*** (KUN-HKAS 155091, holotype). (**a**) Basidia; (**b**) basidiospores; (**c**) pleurocystidia; (**d**) cheilocystidia; (**e**) pileipellis; (**f**) stipitipellis. Scale bars: (**a**,**d**–**f**) = 20 μm; (**b**) = 10 μm; (**c**) = 10 μm.

**Notes**: Morphologically, *L. yadongensis* is easily confused with *L. anthracina* K. Wang, G.J. Li, Z. Du & T. Z. Wei and *L. negrimarginata* A.W. Wilson & G.M. Muell., as all three share dark-toned basidiomata. However, *L. anthracina* has significantly larger basidiomata (pileus diameter 25–65 mm vs. 5–30 mm), sparser lamellae, pileipellis composed of interwoven radiating hyphae with erect terminal hyphal ends, and no cystidia observed [45]. *Laccaria negrimarginata* has smaller pileus (5–15 mm vs. 5–30 mm) with an umbo, dark scales on the surface, black lamellar edges, a longer stipe (30–70 mm) with black streaks, and lacks pleurocystidia and cheilocystidia [23].

**Figure 8:**
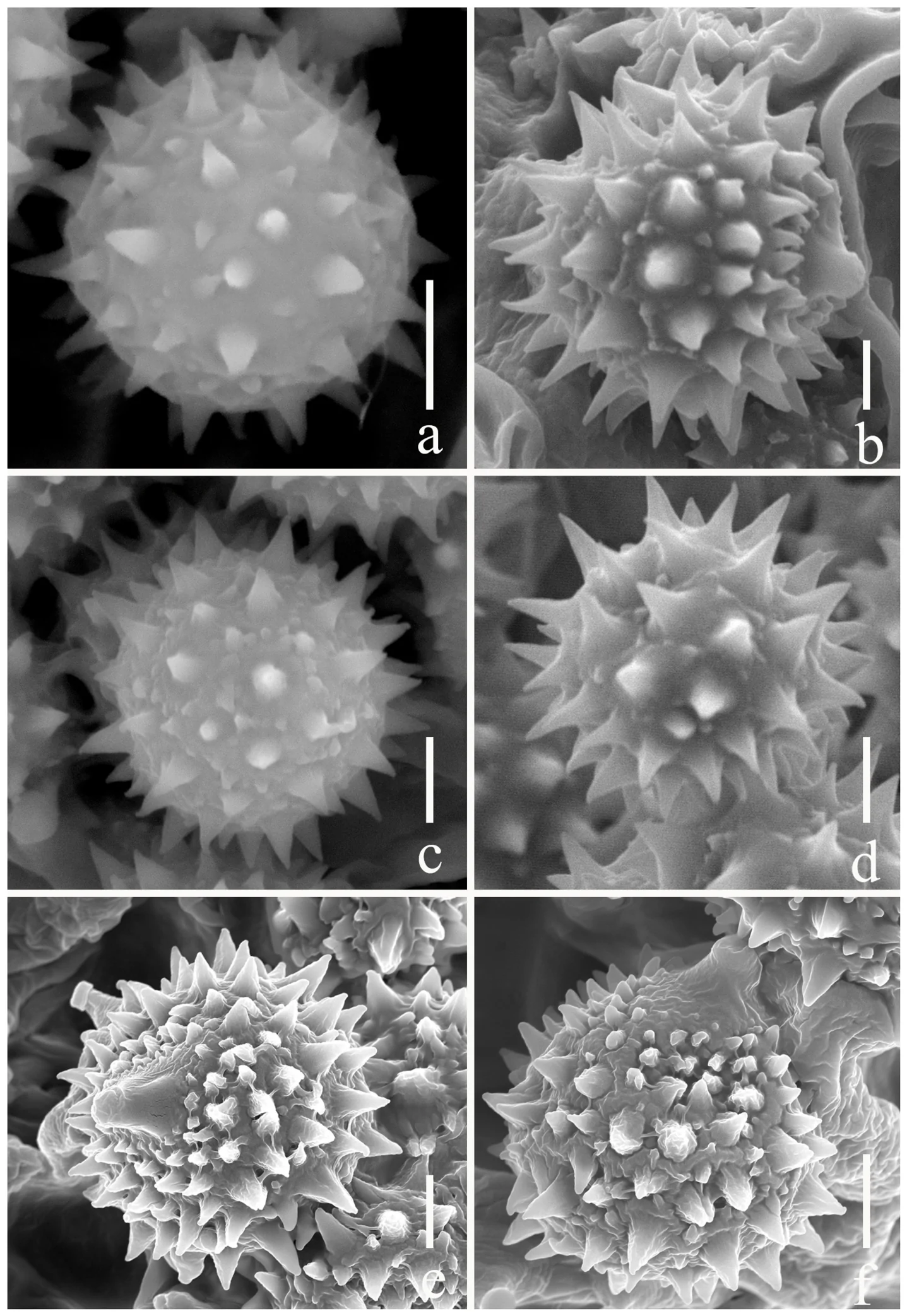
Characteristics of basidiospore ornamentations under scanning electron microscope (SEM). (**a**) *L. aurantirufescens*; (**b**) *L. bisporus*. (**c**) *L. jilongensis*; (**d**) *L. longicystidiata*; (**e**,**f**) *L. yadongensisis*. Scale bars: (**a**–**f**) = 2 μm.

Figure 2m–o, Figure 7 and Figure 8e,f

**Chinese Name**: 亚东蜡蘑 (Ya Dong La Mo) 

**Fungal Names number:** FN 573933

**Diagnosis.** *Laccaria yadongensis* is characterized by small to medium-sized basidiomata; pileus pinkish-brown to fleshy-brown, darker at the center; basidia 4-spored, sterigmata up to 10 μm long; pileipellis composed of inflated hyphae (8–18 μm wide), some with brown pigmentation. Phylogenetically, this species differs from the closely related species *L. jilongensis* (HKAS 155089) by 10.68% (56/524) in ITS sequence divergence.

**Etymology.** The epithet ‘*yadongensis*’ refers to the collection locality of the holotype specimen.

**Holotype**. China, Xizang Autonomous Region, Xigaze City, Yadong County, 28°07′20.40″ N, 89°31′31.29″ E, elev. 3861 m, 24 July 2025, G. Zhao & S. M. Tang, 20250724-107 (KUN-HKAS 155091).

**Description. *Basidiomata*** small to medium-sized. ***Pileus*** 5–30 mm in diam., hemispherical when young, becoming plano-convex to applanate with age, slightly depressed at the center, brownish-black (#2a1e18), pinkish-brown (#bc8f8f) to fleshy-brown (#c19a6b), darker brown (#8b4513) at the center, gradually fading toward the margin, striate on the surface, smooth, shiny, margin very thin, undulate; pileus context thin, below 1–3 mm thick. ***Lamellae*** subdense to crowded, arcuate, adnate, light pinkish-brown (#d9b8b8), 1–3 mm wide; lamella edge even or entire, sometimes undulate; lamellulae in 2–4 tiers. ***Stipe*** 15–50 × 2–6 mm, cylindrical, central, fistulose, equal in width and enlarged at the base, nearly subclavate, concolorous with pileus or paler, surface with longitudinal fibrous striations, with a small amount of white (#ffffff) mycelium attached at the base. ***Odor*** and taste not observed.

***Basidia*** 28–45 × 13–16 μm, av. 38.1 ± 3.8 × 14.9 ± 0.8 μm, clavate, 4-spored; sterigmata 4–10 μm long. ***Basidiospores*** (excluding ornamentation) [150/3/3] 7.3–9.9 × 7.1–9.4 μm, av. 8.4 ± 0.7 × 8.3 ± 0.6 μm, Q_m_ = 1.0–1.1, Q_av._ = 1.01 ± 0.02, subglobose to globose, hyaline, echinulate, spines 2–4 μm long, 1–2 μm wide at the base, crowded. ***Cheilocystidia*** 25–34 × 2–7 μm, av. 29.5 ± 2.2 × 4.0 ± 0.9 μm, narrowly clavate, flexuose, thin-walled, colorless and hyaline, abundant. ***Pleurocystidia*** 14–24 × 3–6 μm, av. 18.5 ± 2.9 × 3.5 ± 0.4 μm, subclavate, narrowly clavate, narrowly utriform, thin-walled, hyaline. ***Lamellar trama*** 60–115 μm thick, regular, composed of slightly thick-walled, filamentous hyphae 2–8 μm wide. ***Lamellar edge*** with a greater number of sterile basidia, composed of clavate, cylindrical inflated cells 6–18 × 4–12 μm, thin-walled, colorless, similar in shape to basidioles. ***Subhymenium*** 10–15 μm thick, tightly interwoven, composed of fusiform or irregular cells, 2–9 × 2–7 μm. ***Pileipellis*** 40–100 μm thick, colorless and hyaline in 5% KOH, composed of inflated or swollen hyphal cells; hyphae 8–18 μm wide, clamped, mostly hyaline but some with brown pigmentation. ***Stipitipellis*** composed of appressed, parallel, simply septate, thick-walled hyphae 5–8 μm wide; stipe trama composed of longitudinally arranged cells, colorless in KOH, with clavate terminal cells, infrequently branching, septate, thick-walled, hyaline hyphae 3–9 μm wide. ***Caulocystidia*** absent. ***Clamp connections*** present at some septa in pileipellis, lamellae, and stipitipellis.

**Habitat and phenology.** Growing gregariously under mixed forests.

**Additional specimens examined.** China, Yunnan Province, Dali Bai Autonomous Prefecture, Cangshan Mountain, elev. 2876 m, 26 September 2020, S. M. Tang, 2020092607 (KUN-HKAS 155092); ibid., elev. 2965 m, 26 September 2020, S. M. Tang,2020092608 (KUN-HKAS 155093).

Multi-locus phylogenetic analysis (Figure 1) indicates that *L. yadongensis* is closely related to *L. jilongensis*. However, *L. jilongensis* has significantly larger basidiomata (pileus diameter 5–110 mm vs. 5–30 mm; stipe length 50–150 mm vs. 15–50 mm), an orange-red pileus, and a pileipellis composed of appressed, parallel, cylindrical filamentous hyphae, 60–120 μm thick. The ITS sequence divergence between *L. yadongensis* (HKAS 155091) and *L. jilongensis* (HKAS 155089) is 10.68% (56/524).

## 4. Discussion

*Laccaria* species not only possess important ecological functions but also demonstrate considerable economic and scientific value in terms of edibility, medicinal properties, and model organism research [64]. A revised checklist of edible fungi in China compiled by Dai et al. (2010) included 11 species of *Laccaria* [65]. The basidiomata of some species in this genus are commonly found in local markets [1,18,45], among which *L. aurantia* Popa, Rexer, Donges, Zhu L. Yang & G. Kost, *L. bicolor* (Maire) P.D. Orton, *L. moshuijun* Popa & Zhu L. Yang, and *L. vinaceoavellanea* Hongo are commercially cultivated and sold as wild edible mushrooms in Sichuan Province, China [18,66], prized for both their palatable flavors and health benefits [1,65]. *Laccaria yunnanensis*, locally known as ‘Pi Tiao Jun’ in southwestern China, is also favored by local residents for its distinctive taste [1]. Furthermore, Tang et al. (2025) reported that the basidiomata of *L. fengkaiensis* Fang Li and other species are highly regarded for their culinary value [31]. In addition to their edible uses, *L. amethystine* Cooke, *L. proxima* (Boud.) Pat., and *L. laccata* (Scop.) Cooke also possess medicinal properties [67]. Moreover, *L. bicolor* and *L. proxima* can grow rapidly under laboratory conditions and are therefore frequently employed as model fungi in studies of fungus–plant symbiotic relationships [33,68,69].

Among the materials used in this study, *Laccaria aurantirufescens* was collected from Yunnan Province, China; *L. bisporus* and *L. longicystidiata* were collected from Thailand; and *L. jilongensis* and *L. yadongensis* were collected from the Xizang Autonomous Region, China. Yunnan is one of the most important biodiversity hotspots in Asia [70], and members of *Laccaria* are commonly found in its forests [46,71]. The Xizang Autonomous Region, characterized by complex geological changes, unique climatic conditions, and diverse ecological environments, is not only a global biodiversity hotspot but also harbors extremely rich macrofungal resources [13,23,45]. Thailand is likewise renowned as a globally significant biodiversity hotspot with high fungal diversity [72,73]. Despite the recognized richness of these regions, the research on species diversity within *Laccaria* remains poorly understood.

Although preliminary phylogenetic analyses have been conducted on some species of *Laccaria*, several critical issues persist [26,31,41,74]. Currently, the available molecular sequence data are insufficient to accurately reconstruct interspecific relationships within the genus [3,18,40]. From 2013 up to 2025, over 90% of new species described based on molecular and morphological characteristics relied solely on ITS sequences, given the limited resolution of this single marker, which often fails to distinguish closely related species or resolve species complexes [23,25,31]. It is urgent to incorporate more protein-coding genes to further clarify interspecific phylogeny. Additionally, most traditionally recognized species lack molecular data from their type specimens, leaving their phylogenetic positions uncertain and severely hindering taxonomic progress. For instance, the holotype specimens of *L. amethystea* (Bull.) Murrill and *L. bisporigera* Contu & Ballero lack sequence data, making it difficult to determine their affinities with other lineages within *Laccaria*. In contrast, an epitype has been designated for *L. laccata* [21], providing phylogenetic data to clearly circumscribe this species and enabling reliable comparisons with other *Laccaria* taxa [19,63,75,76]. Moreover, previous infrageneric classifications, which divided *Laccaria* into two subgenera and seven sections based on morphology, have not been substantiated by molecular phylogenetics [18,35,77,78,79].

In view of these challenges, future research should focus on the following aspects. First, expanded sampling in understudied regions, particularly in Asia, is essential to uncover global species diversity; targeted surveys in the tropical and subtropical forests of Southeast Asia, which are rich in fungi but remain underexplored, would help fill biogeographic gaps and shed light on the evolutionary origins and dispersal patterns of the genus. Second, the application of innovative techniques such as genome skimming should be employed to obtain molecular data from type specimens, thereby clarifying the phylogenetic positions of historically recognized taxa. This approach can efficiently generate high-quality sequences from aged or preserved material, resolving the long-standing issue of missing molecular data for traditional species and enabling a robust revision of *Laccaria* taxonomy. Crucially, because *Laccaria* species form ectomycorrhizal symbioses with a wide range of woody plants, playing a vital role in nutrient cycling and forest ecosystem stability, a well-resolved phylogenetic framework and expanded species diversity data will provide the foundation for future investigations into the specificity of these symbioses and the ecological adaptability of *Laccaria* species across different habitats.

## Figures and Tables

**Table 1:** Lists the names, specimen numbers, collection localities, and corresponding GenBank accession numbers for the ITS, LSU, *rpb*2, and *tef*1-α sequences of the *Laccaria* taxa used in this study, along with relevant references. Species newly described in this study are indicated in bold, and an asterisk (*) following the specimen number denotes the holotype specimen.

Species Name	Sample No.	Location	GenBank Accession				References
			ITS	LSU	*rpb*2	*tef*1	
*Laccaria acanthospora*	HKAS45998	China	KU685719	KU685870	KU686069	-	[23]
*L. acanthospora*	AWW485 *	China	JX504102	JX504186	-	-	[23]
*L. acanthospora*	HKAS147006	China	PV927340	PV857763	PX943789	-	Direct Submission
*L. affinis*	GDOR5565 (epitype)	England	PQ642690	-	PQ653981	-	[20]
*L. affinis*	GDOR5564	England	PQ642691	-	-	-	[20]
*L. alba*	AWW438	China	JX504094	JX504178	KU685912	KU686072	[23]
*L. alba*	TPML20120807-69	South Korea	MG519542	MG519583	MG551616	MG551649	[40]
*L. albifolia*	GDOR5569 *	Italy	PQ642680	PQ642694	-	PQ653979	[20]
*L. albifolia*	GDOR 5573	Italy	PQ642683	PQ642696	PQ653980	PQ653978	[20]
*L. albostipitata*	HKAS 123300 *	China	PQ651565	PQ753313	PQ753328	PQ753342	[28]
*L. albostipitata*	HKAS 123285	China	PQ651566	PQ753314	PQ753329	PQ753343	[28]
*L. ambigua*	PDD89696 *	New Zealand	KU685725	KU685876	KU686018	KU686132	[41]
*L. amethysteo-occidentalis*	AWW556	America	JX504107	JX504191	KU685919	-	[23]
*L. amethysteo-occidentalis*	KG P40 *	America	DQ822817	-	-	-	[23]
*L. amethystina*	GMM7633	France	JX504154	JX504228	-	-	[41]
*L. amethystina*	GMM7621	France	JX504150	JX504224	KU686046	KU686152	[23]
*L. angustilamella*	BAP226	China	JX504118	JX504201	-	-	[23]
*L. anthracina*	HMAS254678 *	China	KX496973	-	-	-	[45]
*L. anthracina*	HMAS260494	China	KX496974	-	-	-	[45]
*L.araneosa*	SFC2013091721 *	South Korea	MG519549	MG519589	MG551622	MG551655	[43]
*L. araneosa*	TPML20120912-40 *	South Korea	MG519548	MG519588	MG551621	MG551654	[43]
*L. aurantia*	KUNF78557 *	China	NR154113	-	-	-	[46]
*L. aurantia*	MBFB001109	Japan	JQ681209	-	-	-	[46]
*L. aurantiaca*	HKAS 123244 *	China	PQ651572	PQ720997	PQ753335	PQ753349	[28]
*L. aurantiaca*	HKAS 123246	China	PQ651573	PQ720998	PQ753336	PQ753350	[28]
***L. aurantirufescens***	HKAS 151666 *	**China**	**PZ092816**	**PZ102785**	**PZ197999**	-	**This study**
***L. aurantirufescens***	**HKAS 151667**	**China**	**PZ092855**	**PZ102456**	**PZ197998**	**PZ198004**	**This study**
*L. bicolor*	HKAS44062	China	JX504159	JX504235	KU686068	-	[23]
*L. bicolor*	KA130253	South Korea	MG519524	MG519570	MG551599	MG551636	[43]
***L. bisporus***	MFLU23-0328 *	**Thailand**	**PZ092895**	**PZ102459**	**PZ198002**	**PZ198007**	**This study**
***L. bisporus***	**MFLU23-0329**	**Thailand**	**PZ092898**	**PZ102460**	**PZ198003**	-	**This study**
*L. bullipellis*	AWW465 *	China	JX504100	JX504184	KU685914	-	[23]
*L. brunnea*	HKAS 123286 *	China	PQ651574	PQ721003	PQ753337	PQ753351	[28]
*L. brunnea*	HKAS 144550	China	PQ651578	PQ721007	PQ753341	PQ753355	[28]
*L. canaliculata*	GMM7267	Australia	JX504137	JX504213	KU685960	KU686093	[23]
*L. canaliculata*	GMM7251	Australia	KU685669	KU685812	KU686090	KU685955	[23]
*L. carminostipes*	MHHNU 11944	China	PV300443	PV300522	PV467480	PV339966	[19]
*L. carminostipes*	MHHNU 31553 *	China	PV300441	-	PV467478	PV339964	[19]
*L. cinnabarina*	KUN-HKAS79704	China	OR722589	OR722600	PP171547	PP171557	[62]
*L. cinnabarina*	KUN-HKAS80885 *	China	OR722587	OR722595	-	-	[62]
*L. daliensis*	HKAS147004 *	China	PV850075	PV857700	PX425769	-	Direct Submission
*L. daliensis*	HKAS147005	China	PV856217	PV857614	PX943787	PX607686	Direct Submission
*L. daliensis*	HKAS 147007	China	PX380966	PV857675	PX943788	PX607685	Direct Submission
*L. dallingii*	Corrales 543	Panama	MT279238	MT279213	MT431187	MT436076	[16]
*L. dallingii*	Corrales 571 *	Panama	MT279240	MT279214	-	-	[16]
*L. darjeelingensis*	CUHAM788 *	India	OQ607624	-	-	-	[19]
*L. fagacicola*	HKAS90435 *	China	MW540806	-	-	-	[26]
*L. fagacicola*	HKAS107731	China	MW540807	-	-	-	[26]
*L. fengkaiensis*	HKAS106739 *	China	MN585657	MN621238	-	-	[44]
*L. fengkaiensis*	HKAS106741	China	MN585658	-	-	-	[44]
*L. fortunensis*	Corrales 74 *	Panama	MT279246	-	-	-	[16]
*L. fortunensis*	Corrales 75	Panama	MT279247	-	-	-	[16]
*L. fulvogrisea*	KUN-F78556 *	China	NR154114	-	-	-	[46]
*L. fulvogrisea*	KUN-FB-101105	China	JQ681210	-	-	-	[46]
*L. gomezii*	F1102433	CostaRica	-	MT279205	MT431180	-	[16]
*L. gomezii*	GMM7173	CostaRica	MT279227	MT279207	MT431182	MT436071	[16]
*L. griseolilacina*	SFC20190919-48 *	South Korea	MT322981	MT322983	MT333269	MT333266	[30]
*L. guizhouensis*	HMAS352265 *	China	OP244890	-	-	-	[42]
*L. guizhouensis*	HMAS352266	China	OP244891	-	-	-	[42]
*L. galerinoides*	F1081213	Chile	KU685634	KU685778	KU686078	KU685929	[41]
*L. galerinoides*	F1080983	Argentina	KU685632	KU685776	KU686077	KU685927	[41]
*L. glabripes*	GMM7521	New Zealand	KU685708	KU685849	KU685991	KU686117	[41]
*L. glabripes*	GMM7534	New Zealand	KU685711	KU685852	-	-	[41]
*L. himalayensis*	AWW463	China	JX504098	JX504182	KU685913	-	[23]
*L. himalayensis*	AWW484 *	China	JX504101	JX504185	KU685915	-	[23]
*L. indohimalayana*	KD 17-46	India	MK575505	-	-	-	Direct Submission
*L. indohimalayana*	KD 17-20	India	MK584157	-	-	-	Direct Submission
*L. japonica*	F64167 *	Japan	KU962988	-	-	-	[47]
*L. japonica*	SFC2012072212	South Korea	MG519518	MG519566	MG551595	MG551633	[43]
***L. jilongensis***	**HKAS 155090**	**China**	**PZ2493108**	**PZ502806**	**PZ527864**	**PZ538357**	**This study**
***L. jilongensis***	HKAS 155089 *	**China**	**PZ2493109**	**PZ502808**	**-**	**PZ538358**	**This study**
*L. longipes*	F1092175	America	KU685637	KU685780	-	-	[41]
*L. longipes*	MQ18R253-QFB30769	Canada	MN992191	-	-	-	Direct Submission
*L. laccata*	C4083, BAFC (epitype)	Sweden	PV700553	PV700591	PV835003	-	[21]
*L. laccata*	GDOR5794	England	PV700554	-	PV835008	-	[21]
*L. laccata*	GDOR5788	Italy	PV700555	PV700592	PV835004	-	[21]
*L. lateritia*	RGB 166658	Malaysia	JN235950	-	-	-	[41]
*L. lateritia*	RGB 166659	Malaysia	JN235949	-	-	-	[41]
*L. longistriata*	KUN-HKAS123801 *	China	OQ396730	-	OR347685	-	[62]
*L. longistriata*	KUN-HKAS115989	China	OQ396729	-	OR347682	OR347688	[62]
***L. longicystidiata***	MFLU23-0336 *	**Thailand**	**PZ092861**	**PZ102786**	-	-	**This study**
***L. longicystidiata***	**MFLU23-0335**	**Thailand**	**PZ092862**	**PZ102457**	**PZ198000**	**PZ198006**	**This study**
***L. longicystidiata***	**MFLU23-0334**	**Thailand**	**PZ092864**	**PZ102458**	**PZ198001**	**PZ198005**	**This study**
*L. macrocystidiata*	GDOR5075 (epitype)	Italy	MW584890	-	-	-	Direct Submission
*L. mangshanensis*	MHHNU 8856	China	PV300438	PV300519	PV467476	PV339962	[19]
*L. mangshanensis*	MHHNU 8850 *	China	PV300437	PV300518	PV467475	PV339961	[19]
*L. miniata*	GDGM76043 *	China	OR689440	OR785476	-	-	[42]
*L. montana*	C5442 *	Switzerland	OR419936	-	-	-	Direct Submission
*L. montana*	M5464	Switzerland	OR419935	-	-	-	Direct Submission
*L. moshuijun*	HKAS 93732 *	China	KU962989	-	-	-	[47]
*L. moshuijun*	MB-001113	China	KU962985	-	-	-	[47]
*L. murina*	ASIS216	South Korea	MG519553	-	-	-	[43]
*L. murina*	ASIS24249	South Korea	MG519552	MG519592	MG551625	MG551658	[43]
*L. nanlingensis*	GDGM 84954 *	China	OR689442	OR785478	OR835199	OR826273	[18]
*L. nanlingensis*	GDGM 84949	China	OR689441	OR785477	OR835198	OR826274	[18]
*L. negrimarginata*	GMM7631	France	JX504152	JX504226	-	-	[23]
*L. neovinaceoavellanea*	GDGM52852 *	China	OR689447	OR785479	-	-	[18]
*L. neovinaceoavellanea*	GDGM53063	China	OR689448	OR785480	-	-	[18]
*L. nitrophila*	Corrales 467	Panama	MT279233	-	-	-	[16]
*L. nitrophila*	Corrales 595 *	Panama	MT279236	MT279211	MT431186	MT436074	[16]
*L. nobilis*	F1091206	America	KU685636	KU685779	-	-	[41]
*L. nobilis*	AWW584	America	JX504110	JX504193	KU685922	-	[23]
*L. ohiensis*	KH-07192006-1	USA	KU685720	KU685871	KU686014	-	[41]
*L. ohiensis*	GMM7028	Russia	KU685653	KU685796	KU685939	-	[41]
*L. oblongospora*	ObiFr	France	GQ406466	-	-	-	[63]
*L. ochropurpurea*	PRL4777	America	KU685733	KU685883	KU686025	-	[41]
*L. pallidorosea*	KUN-HKAS53170	China	MW540809	-	-	-	[26]
*L. pallidorosea*	KUN-HKAS107730 *	China	MW540808	-	-	-	[26]
*L. parva*	SFC20120919-05 *	Korea	MG519529	MG519573	MG551604	MG551640	[43]
*L. parva*	SFC20120906-01	Korea	MG519527	MG519572	MG551639	MG551602	[43]
*L. pakistanica*	LAH38689 *	Pakistan	PV577726	PV600478	-	-	[22]
*L. pakistanica*	LAH38690	Pakistan	PV577725	-	-	-	[22]
*L. populina*	GDOR411 *	Italy	MN871894	MN873018	-	-	Direct Submission
*L. populina*	GDOR 408	Italy	MN871895	MN873017	-	-	Direct Submission
*L. prava*	HKAS106742 *	China	MN585660	-	-	-	[44]
*L. prava*	HKAS106745	China	MN585661	-	-	-	[44]
*L. proxima*	F1081079	Argentina	KU685633	KU685777	KU685928	-	[41]
*L. proxima*	GMM7584	Russia	KU685717	KU685858	KU685999	KU686120	[41]
*L. pseudoalba*	MFLU 22-0106 *	Thailand	ON557377	ON556492	ON598886	-	[27]
*L. pseudoalba*	HKAS 110664	Thailand	ON557376	ON556491	ON598887	ON598894	[27]
*L. pseudomontana*	Cripps 1771	America	DQ149870	-	-	-	[30]
*L. pseudomontana*	pse1625 *	America	DQ149871	-	-	-	[30]
*L. pumila*	pum1252	America	DQ149864	-	-	-	[30]
*L. aff. montana*	AWW446	China	JX504097	JX504181	KU686054	KU686157	[23]
*L. pumila*	HKAS147008	China	PV927342	PV866769	PX943783	-	Direct Submission
*L. pumila*	HKAS147009	China	PV927341	PV866770	PX943784	-	Direct Submission
*L. roseoalbescens*	LM5099 *	Mexico	KJ874328	KJ874331	-	-	[24]
*L. roseoalbescens*	VB4678	Mexico	KJ590509	KJ590510	-	-	[24]
*L. rubra*	HKAS 123291 *	China	PQ651570	PQ776317	PQ753333	PQ753347	[28]
*L. rubra*	HKAS 123292	China	PQ651571	PQ776318	PQ753334	PQ753348	[28]
*L. rubroalba*	KUN-HKA 90753 *	China	KX449358	-	-	-	[25]
*L. rubroalba*	KUN-HKA 90758	China	KX449357	-	-	-	[25]
*L. rufobrunnea*	GDGM82878 *	China	OR689443	OR785482	OR835197	OR826272	[18]
*L. rufobrunnea*	GDGM89627	China	OR689444	OR785483	-	-	[18]
*L. salmonicolor*	GMM7596 *	China	JX504143	JX504218	KU686045	KU686151	[23]
*L. salmonicolor*	GMM7602	China	JX504145	-	-	-	[23]
*L. sinolateritia*	MHHNU 11958	China	PV300445	PV300524	PV467482	PV339968	[19]
*L. sinolateritia*	MHHNU 11956 *	China	PV300444	PV300523	PV467481	PV339967	[19]
*L. spinulosa*	KUN-HKAS129615 *	China	OR722591	OR722598	PP171551	-	[62]
*L. spinulosa*	KUN-HKAS90444	China	OR722590	OR722597	PP171553	-	[62]
*L. stellata*	Corrales27	Panama	MT279231	MT279210	-	MT431185	[1]
*L. stellata*	SYC 207	Panama	KP877339	-	-	-	[31]
*L. striatula*	1475	USA	OQ612526	-	-	-	[19]
*L. striatula*	CNV105	unknown	MT345281	-	-	-	Direct Submission
*L. squarrosa*	DM63 *	Mexico	MF669958	MF669965	-	-	[19]
*L. squarrosa*	DM121	Mexico	MF669960	MF669967	-	-	[19]
*L. subroseoalbescens*	MFLU23-0339 *	Thailand	PP785397	PP789598	-	-	[27]
*L. subroseoalbescens*	MFLU23-0340	Thailand	PP785398	PP789599	-	-	[27]
*L. torosa*	SFC2015090217 *	South Korea	MG519561	MG519598	MG551631	MG551664	[43]
*L. torosa*	KA12-1306	South Korea	MG519562	-	-	-	[43]
*L. tortilis*	ASIS22273 *	South Korea	MG519533	MG519576	MG551608	MG551644	[43]
*L. tortilis*	GMM7635	France	JX504155	KU685906	KU686053	KU686156	[23]
*L. trichodermophora*	F1111951	Costa Rica	KU685640	KU685784	KU686063	-	[41]
*L. trichodermophora*	GMM7733	America	JX504157	-	KU686013	-	[23]
*L. trichodermophora*	TENN42523 *	USA	DQ149868	-	-	-	[30]
*L. trullisata*	PRL7587	China	JX504170	JX504247	KU686047	KU686153	[23]
*L. trullisata*	WCG2075	unknown	KM067894	-	-	-	[41]
*L. umbilicata*	GDGM82883	China	OR689445	OR785485	OR835194	OR826270	[18]
*L. umbilicata*	GDGM82911 *	China	OR689446	OR785486	OR835192	OR826268	[18]
*L. versiforma*	KNU2012100803	South Korea	MG519560	MG519597	MG551630	MG551663	[43]
*L. versiforma*	SFC20120926-01 *	South Korea	MG519556	MG519594	MG551627	MG551660	[43]
*L. violaceonana*	HKAS123243 *	China	PV857581	PV857703	-	-	Direct Submission
*L. violaceonana*	HKAS 123290	China	PV857576	PV857701	PX943785	-	Direct Submission
*L. vinaceoavellanea*	A2986	Japan	JN942810	JN939738	JN993520	-	Direct Submission
*L. vinaceoavellanea*	SFC20150810-10	South Korea	MG519539	MG519580	MG551614	MG551646	[43]
*L. violaceonigra*	GMM7520	New Zealand	KU685707	KU685848	KU685990	-	[41]
***L. yadongensis***	HKAS 155091 *	**China**	**PZ2493110**	**PZ502807**	**PZ527865**	**PZ538359**	**This study**
***L. yadongensis***	**HKAS 155092**	**China**	**PZ2493112**	**PZ502803**	**PZ527866**	**PZ538360**	**This study**
***L. yadongensis***	**HKAS 155093**	**China**	**PZ2493111**	**PZ502805**	**PZ527867**	**PZ538361**	**This study**
*L. violaceotincta*	CAL1389 *	India	MK141034	-	-	-	[32]
*L. yunnanensis*	KUNF78558 *	China	NR154115	-	-	-	[46]
*L. yunnanensis*	MFLU 22-0107	Thailand	ON557374	ON556488	-	ON598892	[27]
*Mythicomyces corneipes*	ES11.10.2. A	Germany	KC964108	-	-	-	[31]
*M. corneipes*	AFTOLID972	Germany	DQ404393	AY745707	DQ408110	DQ029197	[31]

Note: The ‘-’ denotes the unavailability of the sequence.

## Data Availability

Data are contained within the article.
